# Understanding the Cross-Talk of Redox Metabolism and Fe-S Cluster Biogenesis in Leishmania Through Systems Biology Approach

**DOI:** 10.3389/fcimb.2019.00015

**Published:** 2019-02-04

**Authors:** Anurag Kumar, Nutan Chauhan, Shailza Singh

**Affiliations:** National Centre for Cell Science, Pune, India

**Keywords:** leishmaniasis, redox metabolism, trypanothione, Fe/S cluster proteins, glutaredoxin, systems biology, kinetic modeling

## Abstract

Leishmania parasites possess an exceptional oxidant and chemical defense mechanism, involving a very unique small molecular weight thiol, trypanothione (T[SH]_2_), that helps the parasite to manage its survival inside the host macrophage. The reduced state of T[SH]_2_ is maintained by NADPH-dependent trypanothione reductase (TryR) by recycling trypanothione disulfide (TS_2_). Along with its most important role as central reductant, T[SH]_2_ have also been assumed to regulate the activation of iron-sulfur cluster proteins (Fe/S). Fe/S clusters are versatile cofactors of various proteins and execute a much broader range of essential biological processes viz., TCA cycle, redox homeostasis, etc. Although, several Fe/S cluster proteins and their roles have been identified in Leishmania, some of the components of how T[SH]_2_ is involved in the regulation of Fe/S proteins remains to be explored. In pursuit of this aim, a systems biology approach was undertaken to get an insight into the overall picture to unravel how T[SH]_2_ synthesis and reduction is linked with the regulation of Fe/S cluster proteins and controls the redox homeostasis at a larger scale. In the current study, we constructed an *in silico* kinetic model of T[SH]_2_ metabolism. T[SH]_2_ reduction reaction was introduced with a perturbation in the form of its inhibition to predict the overall behavior of the model. The main control of reaction fluxes were exerted by TryR reaction rate that affected almost all the important reactions in the model. It was observed that the model was more sensitive to the perturbation introduced in TryR reaction, 5 to 6-fold. Furthermore, due to inhibition, the T[SH]_2_ synthesis rate was observed to be gradually decreased by 8 to 14-fold. This has also caused an elevated level of free radicals which apparently affected the activation of Fe/S cluster proteins. The present kinetic model has demonstrated the importance of T[SH]_2_ in leishmanial cellular redox metabolism. Hence, we suggest that, by designing highly potent and specific inhibitors of TryR enzyme, inhibition of T[SH]_2_ reduction and overall inhibition of most of the downstream pathways including Fe/S protein activation reactions, can be accomplished.

## Introduction

Cutaneous leishmaniasis (CL), the most common form of leishmaniasis, has always been neglected as a major public health problem due to its non-fatality. The severity of the disease is in terms of disfigurement and residual scars. The causative agent of CL, a protozoan parasite, *Leishmania major* has a digenetic lifecycle and lives in two hosts, sandfly and human, in the form of flagellated promastigotes and non-flagellated amastigotes, respectively.

Inside the mammalian host, the parasite lives in the lethal enzymatic environment of macrophage cells, where they have to deal with the macrophage generated oxidative stress to survive. Remarkably, their survival is contributed by a very unique redox metabolism that the parasite has evolved with. The defense machinery of the parasite involves one main central unusual thiol reductant, trypanothione (N1,N8-bis-glutathionylspermidine; T[SH]_2_) (Fairlamb and Cerami, 1985; Fairlamb et al., 1985). T[SH]_2_ is synthesized by a bifunctional trypanothione synthetase (TryS) that covalently attaches two molecules of glutathione (GSH) onto one molecule of spermidine (Spd) in a two-step process. T[SH]_2_ plays a pivotal role in carrying out a number of many important cellular functions, such as detoxification of H_2_O_2_ and metals, drug resistance (e.g., antimonials) (Borst and Ouellette, 1995; Mukhopadhyay et al., 1996; Wyllie et al., 2004) and defense against chemical and oxidant stress, maintaining the redox balance by protein disulfide reduction and indirect synthesis of deoxyribonucleotide (Fairlamb and Cerami, 1985; Krauth-Siegel and Lüdemann, 1996; Flohe et al., 1999; Dormeyer et al., 2001). Further, the reduced state of T[SH]_2_ is maintained by NADPH-dependent trypanothione reductase (TryR), a unique dimeric flavoenzyme, which recycles trypanothione disulfide (TS_2_) back to T[SH]_2_ (Krauth-Siegel et al., 1987; Fairlamb et al., 1989; Nogoceke et al., 1997; Fairlamb, 1999; Montemartini et al., 2000; Hofmann et al., 2001; Flohé et al., 2002).

Previous studies have demonstrated the essentiality of TryR for parasite survival and due to its non-existence in mammals, it has been validated as an attractive therapeutic target (Dumas et al., 1997; Tovar et al., 1998a,b; Krieger et al., 2000; Krauth-Siegel et al., 2003; Krauth-Siegel and Comini, 2008; Holloway et al., 2009). The functional analog enzyme for TryR in a mammalian host is glutathione reductase (GR) (Krauth-Siegel and Inhoff, 2003; Krauth-Siegel et al., 2003). Although, TryR and human GR have similar catalytic mechanisms, they are specific to their respective disulfide substrates (Marsh and Bradley, 1997). Numerous crystallographic studies revealed that TryR remains active in a homodimeric form (Shames et al., 1986; Krauth-Siegel et al., 1987; Baiocco et al., 2013) and the catalytic site is contributed from both the subunits forming two regions, the NADP site (N-site) and the active site (G-site). Interestingly, their crossed complexes, TryR-GSSG and GR-T(S)2 were found non-catalytic (Shames et al., 1986; Krauth-Siegel et al., 1987). Moreover, the binding site in TryR is wider and more hydrophobic exhibiting an overall negative charge (Kuriyan et al., 1991; Hunter et al., 1992; Stoll et al., 1997). All these features raise the possibility to inhibit TR selectively without affecting the host's machinery.

**Figure 1:**
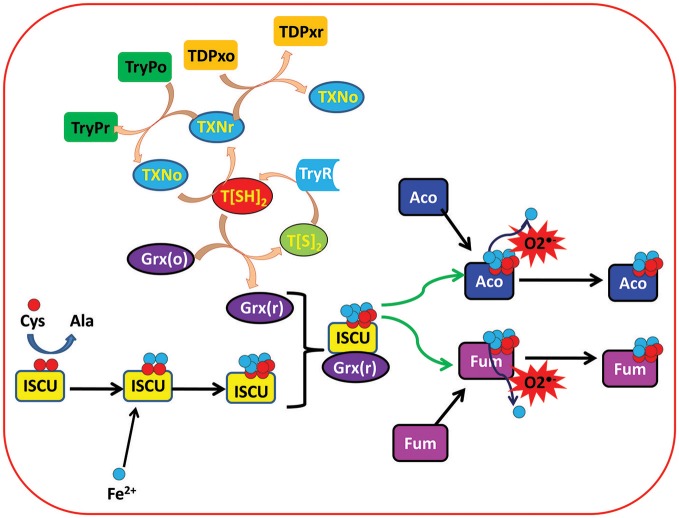
Depicting the important role of trypanothione (T[SH]_2_) in regulation of ISC proteins in *Leishmania* parasite. Trypanothione disulfide (T[S]_2_) is reduced to Trypanothione (T[SH]_2_) with the help of Trypanothione reductase (TryR) enzyme. Reduced trypanothione helps in reduction of different enzymes and proteins including Tryparedoxins (TXN), Tryparedoxin Peroxidases (TryP), Tryparedoxin-dependent Peroxidases (TDPx), and Glutaredoxins (Grx). On the other hand, Iron sulfur cluster unit (ISCU) obtains sulfur (S) and iron (Fe^2+^) from cysteine and extracellular environment, respectively, and binds with reduced Grx to form Grx(r)-ISCU[4Fe-4S] complex. Further, Grx(r)-ISCU[4Fe-4S] activates ISC proteins (Aconitase and Fumarase) by transferring its [4Fe-4S] unit to these proteins/enzymes. Under oxidative stress conditions, free radicals like Superoxide free radicals (${\text{O}}_{2}^{•-}$) reacts with activated ISC proteins/enzymes and leaches out one Fe^+3^ ion. That results in inactivation of the proteins/enzymes.

Other than these two enzymes, a thioredoxin (Trx) like tryparedoxin (TXN) (Qi and Grishin, 2005), an oxidoreductase, is also the main component of the leishmanial redox system that helps in the activation of many important enzymes through electron shuttling from T[SH]_2_ (Nogoceke et al., 1997; Dormeyer et al., 2001). Glutaredoxins (Grxs) are other ubiquitous small thiol-disulfide oxidoreductases that play crucial roles in the redox homeostasis of the cell by participating in a large number of biological processes involving cellular redox and iron sulfur (Fe/S) metabolism. In mammals, Grxs are GSH dependent, however in trypanosomatids it was found that T[SH]_2_ is the main reducing agent for leishmanial Grxs (Figure 1) (Ceylan et al., 2010).

Structurally, on the basis of the presence of a number of cysteine residues in the conserved motif Grxs are classified as: dithiol Grxs (2-C-Grx) containing a C*XX*C active site and monothiol Grxs (1-C-Grx) with C*XX*S motif. 2-C-Grxs play an important role in catalyzing the deglutathionylation of protein disulfides, whereas this function is lacking in 1-C-Grxs (Rahlfs et al., 2001; Tamarit et al., 2003; Deponte et al., 2005; Fernandes et al., 2005; Filser et al., 2008; Johansson et al., 2011). Recently, Fe/S clusters (iron sulfur cluster; ISC) binding ability of Grxs has been identified in trypanosomes including *Leishmania* (Fernandes et al., 2005; Fladvad et al., 2005; Berndt et al., 2007; Picciocchi et al., 2007; Comini et al., 2008; Iwema et al., 2009; Luo et al., 2010; Johansson et al., 2011; Yeung et al., 2011). This ISC binding ability of both the Grxs is not yet utilized in redox metabolism. These generally play an important role in the regulation of their oxidoreductase activity or to facilitate ISC transfer to other acceptor proteins for the biogenesis of Fe/S proteins. ISC are simple protein cofactors with iron-sulfur moieties and are involved in many biological processes (TCA cycle, etc.). To date, four ISC protein systems have been reported including nitrogen fixing (NIF), ISC, cytosolic iron sulfur protein assembly (CIA), and sulfur mobilization (Lill, 2009).

In yeast, Grx3/4 are reported to be involved in iron homeostasis and play an important role as iron sensors in which they are involved in modulation of Fe/S mediated interactions of Grxs with nuclear transcription factors (Li et al., 2009; Mühlenhoff et al., 2010). However, since, trypanosomes are almost lacking transcription factors, it seems highly unlikely for Grxs to play a similar role in these parasites (Clayton and Shapira, 2007).

T[SH]_2_ being the central efficient reducing agent of Grxs, and its indirect involvement in the activation/biogenesis of ISCs, new possibilities of exploring the iron homeostasis and its relation to redox metabolism have opened new insights for unexplored roles of TryR (Figure 1).

Due to unacceptable levels of drug resistance and high toxicity, there are increased demands to explore new drugs and drug targets against CL. From the above discussion, it is perceivable that the redox metabolism of *Leishmania* is an interesting choice of study due to many enzymes of principal importance. Several of them are already being investigated as potential drug targets from a metabolic as well as a druggability point of view (Flohé, 2012; Olin-Sandoval et al., 2012). Nowadays, systems biology approaches (an integration of mathematical methods and computational approaches) are being utilized to study systems dynamics of complex redox metabolic pathways in many organisms, such as, *Escherichia coli, Saccharomyces cerevisiae* (Beiting and Roos, 2007; Toledano et al., 2007; Nielsen and Jewett, 2008; Pillay et al., 2013). Redox systems have been characterized from systems biology perspective using top-down and bottom-up approaches. Moreover, -omics data is also being integrated to construct metabolic kinetic models to explore the complex mechanisms (Pillay et al., 2013; Go et al., 2014).

In the present work, we have tried to elucidate the underlying mechanism of how T[SH]_2_ directly or indirectly affects the cellular redox metabolism of the parasite by playing a role in activation and influencing the ISC protein biogenesis. The complexity of multi-enzymatic network has led us to construct a kinetic model of ISC protein biogenesis-trypanothione-Grxs relationship. Comprehensive analysis of the models has presented interesting insights related to the redox biology of the parasite.

## Materials and Methods

### Kinetic Modeling and Computer Simulation

**Figure 2:**
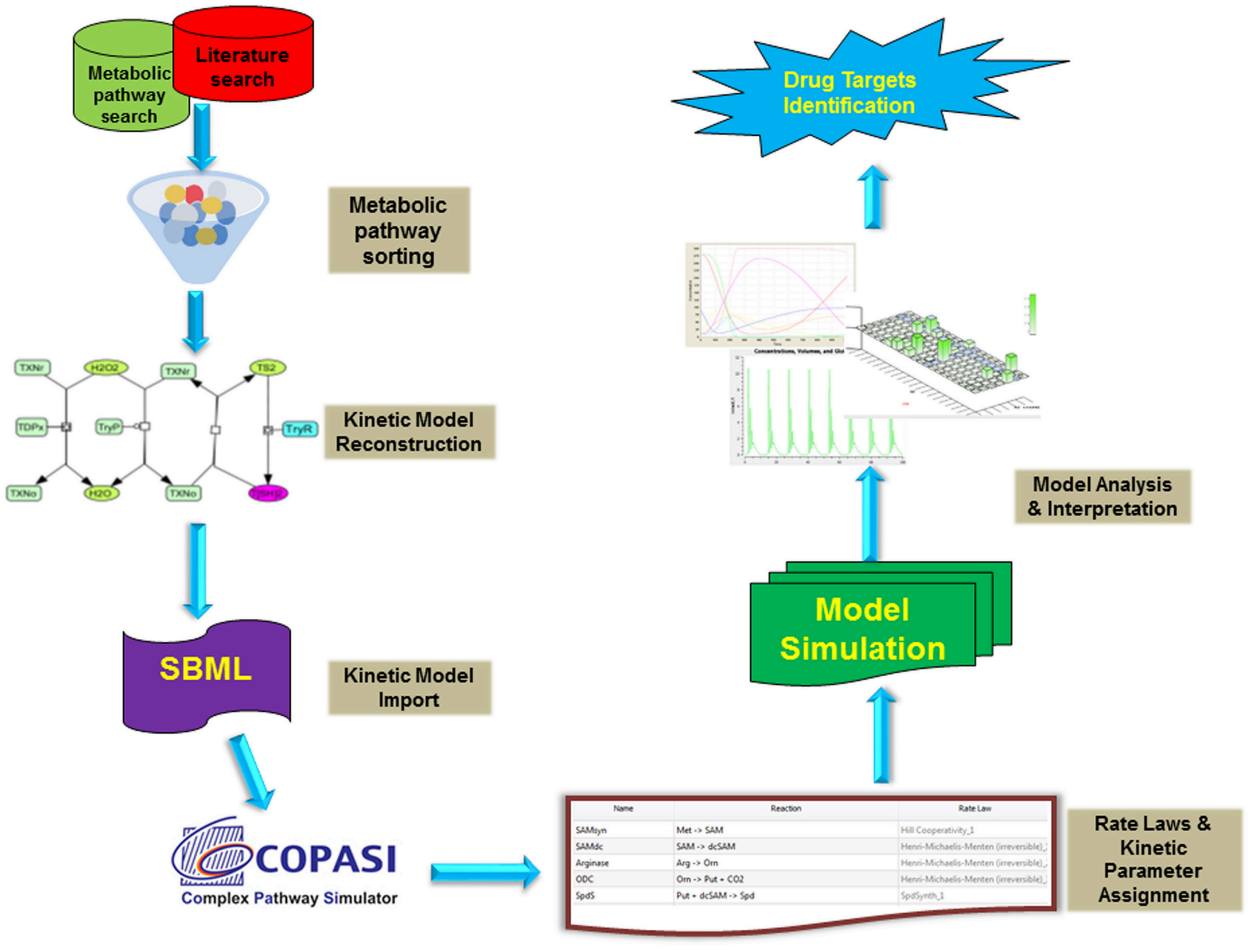
Typical workflow strategies adopted for the kinetic modeling and analysis. Briefly, the metabolic reaction data were retrieved from various databases (LeishCyc, KEGG, etc.) and literature. These pathways were then reconstructed in Cell Designer and exported as SBML model. Further, The SBML model was imported into COPASI and assigned with proper rate laws and parameters followed by its time course kinetic simulation. The model was analyzed and interpreted to propose imported drug targets.

**Figure 3:**
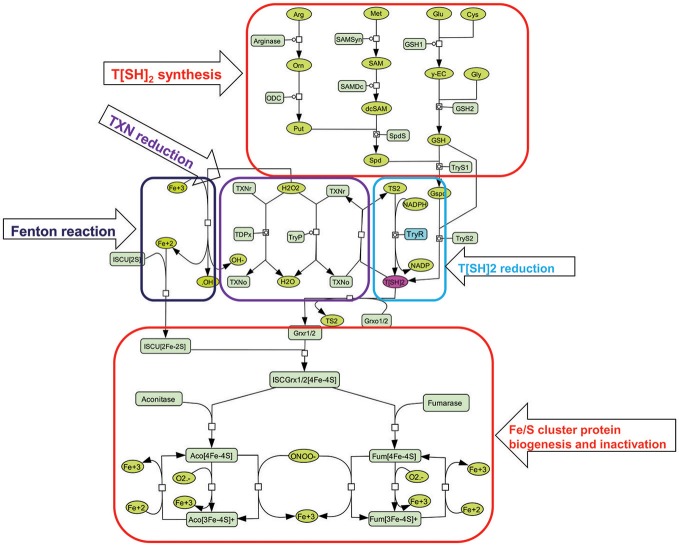
Illustration of kinetic model used for constructing perturbed models. T[SH]_2_ is synthesized from different pathways involving key amino acids [arginine (arg), methionine (met), glutamate (glu), cysteine (cys), glycine (gly)] (*Trypanothione (T[SH]*_2_*) synthesis)*. It is also obtained from the reduction of Trypanothione disulfide (T[S]_2_) with the help of Trypanothione reductase (TryR) enzyme (*T[SH]*_2_ *reduction)*. Furthermore, T[SH]_2_ reduces Tryparedoxins (TXN) that is, further, utilized to neutralize hydrogen peroxide (H_2_O_2_) in Tryparedoxin Peroxidases (TryP) or Tryparedoxin-dependent Peroxidases (TDPx) mediated catalyzed reaction (Tryparedoxin (*TXN) reduction*). This reaction releases free hydroxyl (OH^−^) ion. OH^−^ is then converted to free hydroxyl radical (^•^OH) ions through *Fenton reaction* involving Fe^3+^ ion. On the other hand, Iron sulfur cluster unit (ISCU [4Fe-4S]) is formed (Figure 2), and binds with reduced Grx to form Grx(r)-ISCU[4Fe-4S] complex. Further, Grx(r)-ISCU[4Fe-4S] activates ISC proteins (Aconitase and Fumarase) by transferring its [4Fe-4S] unit to these enzymes. Under oxidative stress conditions, free radicals like Superoxide free radicals (${\text{O}}_{2}^{•}$) and peroxynitrite (ONOO^−^) reacts with activated aconitase[4Fe-4S] and fumarase[4Fe-4S] and inactivates them by removing one Fe^+3^ ion.

All the models were constructed in Cell Designer and exported as Systems biology Markup language (SMBL) file. These SBML files were imported in COPASI version 4.19.140 (Hoops et al., 2006), a simulator for biochemical networks (Figures 2, 3). Each reaction was assigned by appropriate kinetic laws and kept as an irreversible reaction, for uniformity in the model (Table 1, Tables S1, S2). Non-enzymatic reactions were assigned with mass action kinetics, while for enzymatic reactions, Michaelis-Menten rate law; Hill kinetics and Ping-pong kinetics, as applicable, were assigned. The concentrations in the model are in micromolar (μM), and the time unit is in seconds. Fluxes of individual reactions within the model are in μM/s. All the kinetic parameters were obtained (or calculated) from published literature except the hypothetical value of inhibition constant (Ki) for TryR. After model construction, time course simulation and other analyses were performed.

**Table 1:** Basic properties of kinetic models.

	**Model 1**	**Model 2**	**Model 3**	**Model 4**
Reactions	22	29	29	29
Parameters	56	64	64	64
Components	38	45	46	46
Kinetic laws	22	29	29	29

### Parameter Scanning and Sensitivity Analysis

In order to observe the effect of different parameters and concentrations on the model, parameter scanning and sensitivity analysis were performed. The reconstructed network was numerically simulated using Deterministic LSODA ODE solver (Hindmarsh, 1983) for defining the mathematical framework of the model.

## Result and Discussion

Once inside the host macrophage, the survival of leishmanial parasite is mainly contributed by a unique redox metabolism that involves one main low molecular weight thiol molecule, T[SH]_2_ (Fairlamb and Cerami, 1992; Flohe et al., 1999; Dormeyer et al., 2001), that is synthesized by TryS enzyme. However, after performing its function as a main reductant of many enzymes, proteins and other small molecules, it is converted to its oxidized state, TS_2_. To balance between the T[SH]_2_- TS_2_ states, TS_2_ is reduced by an important enzyme, TryR. As mentioned earlier, T[SH]_2_ plays various important roles in a number of processes, such as, regulation of thiol redox balance, etc. Other than these, activation and regulation of Fe/S cluster proteins is another essential task in which T[SH]_2_ has played an important role (Fairlamb and Cerami, 1992; Flohe et al., 1999; Dormeyer et al., 2001) (Figures 1, 3). In order to understand the role of T[SH]_2_, a systems biology approach was adopted through which we tried to get an overall insight of how redox homeostasis is maintained in *Leishmania*.

### Kinetic Model Reconstruction

In our models, we have introduced reactions for the synthesis of T[SH]_2_ via arginine, glycine, glutamate and methionine. The demand for T[SH]_2_ were included in the form of reduction reactions of Grx1/2, TXN, TDPx, and TryP. Grx1/2 is further known to activate other Fe/S cluster proteins (Clayton and Shapira, 2007). In Leishmania, there are many regulatory proteins/enzymes which are activated only after the assembly of Fe/S cluster in their respective binding scaffold. Aconitase and Fumarase are such ISC enzymes containing a cubane [4Fe-4S]^2+^ cluster. These enzymes were taken into account because of their well-established roles in energy metabolism and electron transport chain (ETC) mechanism. We assume, any kind of perturbation in T[SH]_2_ synthesis or reduction might directly affect their activation. Hence, to connect T[SH]_2_ with Fe/S cluster proteins, activation reactions of aconitase and fumarase were added as an important contribution from T[SH]_2_ pathway. Thus, the kinetic model for redox metabolism of *Leishmania* parasite was constructed using the metabolic simulator COPASI (Hoops et al., 2006) (http://www.copasi.org) by applying previously determined rate constants under physiological conditions for substrates of the pathway enzymes (Figure 2). All reactions were considered irreversible and the models were simulated using the LSODA algorithm. The details of the kinetic models are discussed in the sections below.

#### Basal Models

***Model 1***: This basal model has a total of 22 reactions, with 22 kinetic laws, 56 parameters, and 38 components (metabolites) (Figure 3; Table 2). In this model, reactions related to T[SH]_2_ synthesis pathway were included. Further, the demand reactions for T[SH]_2_ were added in the form of Grx1/2, TDPx, TryP, and TXN reduction. The model was further extended and included with Fe/S cluster proteins activation through ISCU-Grx1/2 mediated activators.

**Table 2:** Different rate laws assigned to various reactions in basal kinetic model.

**S.No**.	**Reaction name**	**Reaction**	**Rate Law**
1.	SAMsyn	Met → SAM	Hill Cooperativity
2.	SAMdc	SAM ^→^ dcSAM	Michaelis-Menten
3.	Arginase	Arg ^→^ Orn	Michaelis-Menten
4.	ODC	Orn ^→^ Put + CO_2_	Michaelis-Menten
5.	SpdS	Put + dcSAM ^→^ Spd	Michaelis-Menten
6.	yECS	Glu + Cys ^→^ GluCys	Michaelis-Menten
7.	GS	GluCys + Gly ^→^ GSH	Michaelis-Menten
8.	TryS1	Spd + GSH ^→^ Gspd	Bi substrate Michaelis-Menten
9.	TryS2	Gspd + GSH ^→^ T[SH]_2_	Bi substrate Michaelis-Menten
10.	TryR	TS_2_ + NADPH ^→^ T[SH]_2_ + NADP	Michaelis-Menten
11.	TXNo Reduction	T[SH]_2_ + TXNo ^→^ TXNr + TS_2_	Mass action
12.	TDPx	H_2_O_2_ + TXNr ^→^ TXNo + H_2_O; TDPx	Bi Bi Ping Pong
13.	TryP	H_2_O_2_ + TXNr ^→^ TXNo + H_2_O; TryP	Bi Bi Ping Pong
14.	ISCU activation	2Fe^+2^ + ISCU[2S] ^→^ ISCU[2Fe-2S]	Michaelis-Menten
15.	Aco_activation_grx1	aconitase + ISC-Grx1r[4Fe-4S] ^→^ aconitase[4Fe-4S] + ISCU	Michaelis-Menten
16.	Fum_activation_grx1	Fumarase + ISC-Grx1r[4Fe-4S] ^→^ fumarase[4Fe-4S] + ISCU	Michaelis-Menten
17.	Grx1 reduction	Grx1 + T[SH]_2_^→^ Grx1r + TS_2_	Mass action
18.	Grx2 reduction	Grx2 + T[SH]_2_^→^ Grx2r + TS_2_	Mass action
19.	ISCU-grx1 complex	ISCU[2Fe-2S] + Grx1r ^→^ ISC-Grx1r[4Fe-4S]	Mass action
20.	ISCU-grx2 complex	ISCU[2Fe-2S] + Grx2r ^→^ ISC-Grx2r[4Fe-4S]	Mass action
21.	Aco_activation_grx2	aconitase + ISC-Grx2r[4Fe-4S] ^→^ aconitase[4Fe-4S] + ISCU	Michaelis-Menten
22.	Fum_activation_grx2	fumarase + ISC-Grx2r[4Fe-4S] ^→^ fumarase[4Fe-4S] + ISCU	Michaelis-Menten
23.	Aconitase_${\text{O}}_{2}^{•-}$	${\text{O}}_{2}^{•-}$+ aconitase[4Fe-4S] ^→^ aconitase[3Fe-4S^]+^ + H_2_O_2_ + Fe^+3^	Mass action
24.	Fumarase_${\text{O}}_{2}^{•-}$	${\text{O}}_{2}^{•-}$+ fumarase[4Fe-4S] ^→^ fumarase[3Fe-4S^]+^ + H_2_O_2_ + Fe^+3^	Mass action
25.	Fenton reaction	H2O2 + Fe^+3^^→^OH + OH^−^+ Fe^+2^	Mass action
26.	Fum_activation_Fe^+2^	fumarase[3Fe-4S^]+^ + Fe^+2^^→^ fumarase[4Fe-4S]	Mass action
27.	Aco_activation_Fe^+2^	aconitase[3Fe-4S^]+^ + Fe^+2^^→^ aconitase[4Fe-4S]	Mass action
28.	Aco_ONOO-	aconitase[4Fe-4S] + ONOO^−^^→^ aconitase[3Fe-4S^]+^ + Fe^+3^	Mass action
29.	Fum_ONOO-	fumarase[4Fe-4S] + ONOO^−^^→^ fumarase[3Fe-4S^]+^ + Fe^+3^	Mass action
30.	Perturbed TryR reaction	TS2 -> T[SH]2; drug	Competitive inhibition/non-competitive inhibition

***Model 2***: Another basal model, Model 2, was constructed from Model 1 by connecting it to free radicals' mediated effect on Fe/S proteins. It has been studied that several oxidants such as, hydrogen peroxide, superoxide free radical, and peroxynitrite can inactivate ISC proteins by leaching out one labile iron from the cluster giving [3Fe-4S]^+^ cluster protein. Superoxide free radicals (${\text{O}}_{2}^{•-}$) and peroxynitrite (ONOO^−^) were shown to be particularly reactive with aconitase [4Fe-4S]^2+^ and fumarase [4Fe-4S]^2+^ (Castro et al., 1994; Crack et al., 2014). Rate constants at which ONOO^−^ and ${\text{O}}_{2}^{•-}$ inactivates aconitase are 1.4 **×** 10^5^ M^−1^s^−1^ and 3.5 × 10^6^ M^−1^s^−1^, respectively (Castro et al., 1994). Hence, reactions including ONOO^−^ and ${\text{O}}_{2}^{•-}$ mediated inactivation of aconitase and fumarase were added to Model 1. Further, considering the increased level of free iron after ISC inactivation, fenton reaction was also included in the model (Figure 3, Table 1, Tables S1–S3). As a result, Model 2 consists of a total of 29 reactions with 64 kinetic parameters and 45 metabolites.

#### Perturbed Models

***Model 3 and Model 4***: Modified models were constructed from Model 2 by adding perturbation at TryR reaction in the T[SH]_2_ reduction pathway. A hypothetical inhibitor was used as perturbation whose inhibition rate constant was kept ten times lower than the Km of T[SH]_2_ as per the experimentally determined range (Table S4). To check the effect of TryR inhibition on downstream reactions, competitive and non-competitive inhibition rate laws were assigned to construct ***Models 3*** and ***4***. These models were consisting of 29 reactions with 64 parameters and 46 components (Tables 1, 2).

### Time Course Simulation and Sensitivity Analysis of Kinetic Models

**Figure 4:**
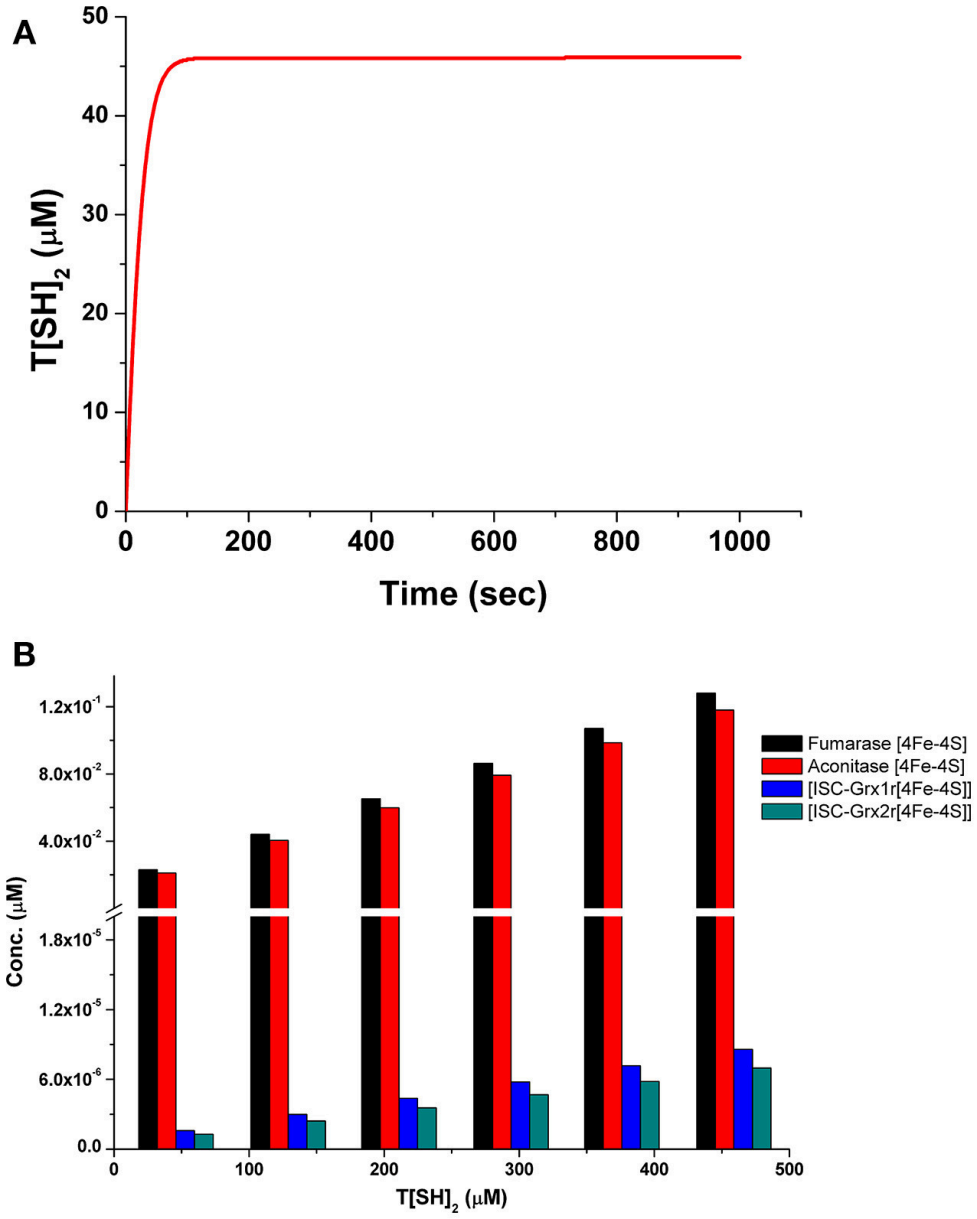
**(A)** Prediction of T[SH]_2_ concentration through time course simulation in basal kinetic model; **(B)** Prediction of the effect of increased concentration of T[SH]_2_ against Fe/S cluster proteins in basal model showing the gradual increase in their concentration.

Time course simulation of basal models for a certain time period showed maximum production of T[SH]_2_ (Figure 4A), that saturated at an early time point in the model. The obtained concentration of T[SH]_2_ was further used to observe how the increasing T[SH]_2_ concentration can affect the level of Fe/S cluster protein activation. Parameter scanning of up to a 10-fold increased T[SH]_2_ concentration against Fe/S cluster proteins in model 1 demonstrated greater effect on Fe/S cluster proteins. The increased concentration of T[SH]_2_ has direct effect on Fe/S proteins, aconitase, and fumarase whose synthesis was gradually increased with the T[SH]_2_ level (Figure 4B) suggesting T[SH]_2_ mediated direct control on the activation of Fe/S proteins.

**Figure 5:**
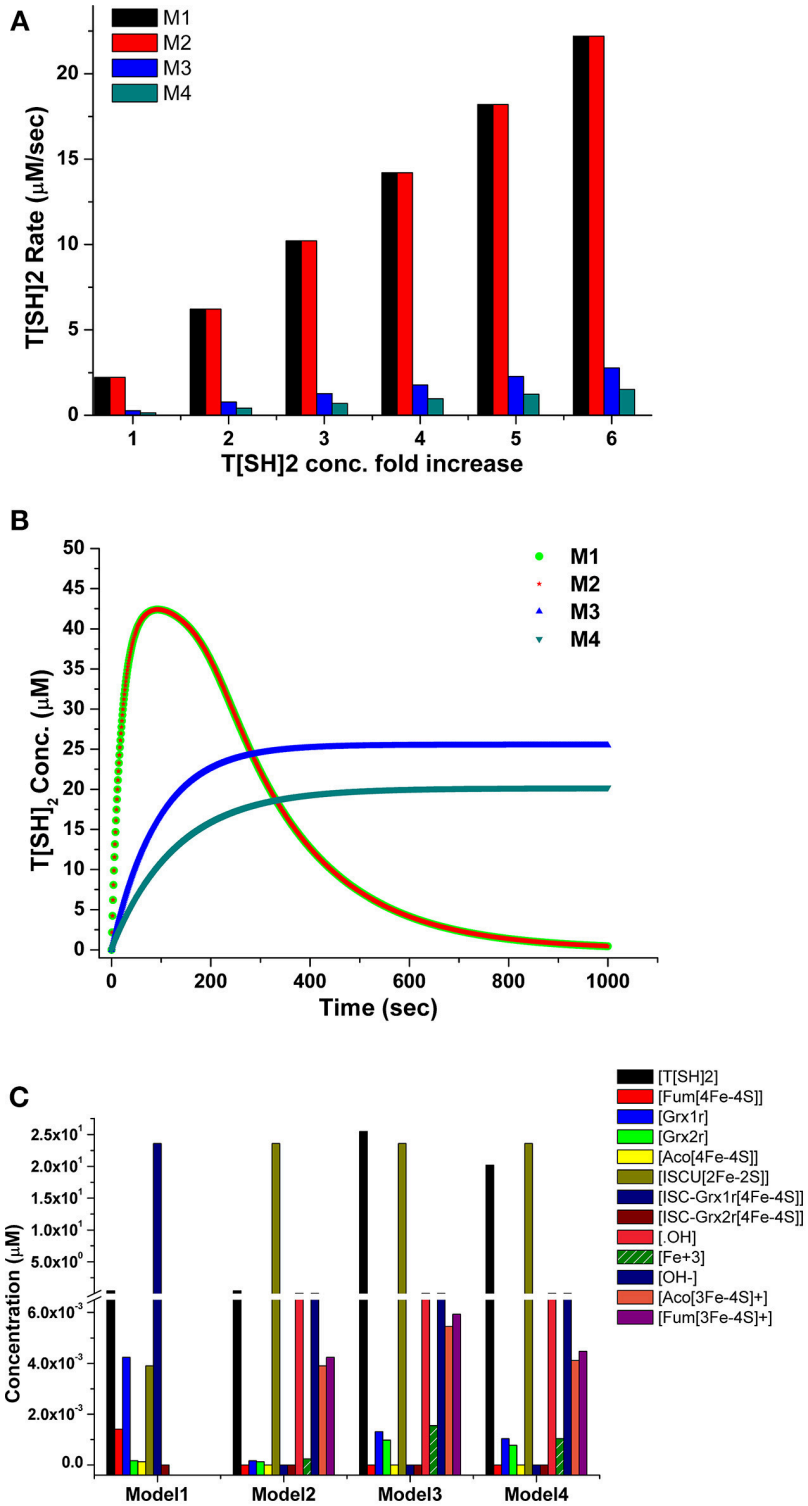
**(A)** Comparison of T[SH]_2_ production rates in four models with respect to the increased concentration of T[SH]_2_. **(B)** Comparison of T[SH]_2_ concentration in four models suggesting the decrease in its level in M3 and M4 models due to applied inhibition kinetics. **(C)** Concentrations of all components were compared in four models showed the level of inactivated aconitase and fumarase and free OH ions was increased.

Comparison of T[SH]_2_ synthesis rates among the models revealed that the synthesis rate was higher in model 1 and model 2 as compared to model 3 and model 4. The rate was gradually decreased, by 8 and 14-folds, in models 3 and 4, respectively, due to perturbed TryR catalyzed reaction (Figure 5A). Similarly, T[SH]_2_ concentrations were also found to be greatly affected due to the perturbation in models 3 and 4 (Figure 5B). Furthermore, other components of the models were also found to be influenced by inhibition applied in respective models (Figure 5C). Through our kinetic models, we were able to predict the effect of inhibition perturbation introduced in the TryR reaction. However, *in vitro* and *in vivo* validation of the same will provide a deeper understanding of the inhibition kinetics. A lot of literature related to the inhibition study of TryR enzyme has already been made available. These studies have already been able to demonstrate the effect of various inhibitors and their inhibition mechanisms against TryR enzyme (Jones et al., 2010; Beig et al., 2015; Saccoliti et al., 2017; Turcano et al., 2018). A previously published kinetic model in *T. cruzi* provided greater insights on the T[SH]2 synthesis and reduction pathways (Olin-Sandoval et al., 2012). They showed the significantly important roles of TryR and other enzymes under oxidative stress conditions and suggested the importance of specificity of the inhibitor against TryR. Through our models along with the previously published inhibition studies, we also suggest that if a TryR specific inhibitor is designed, it could greatly affect the activity of TryR as well as its downstream pathways because of the valuable role of T[SH]_2_.

It is worth mentioning here that, in 4Fe-4S cluster, only three of the iron atoms are attached to the protein, but the fourth iron interacts only with the sulfur of Fe/S cluster. This iron has a free coordination site that helps in its participation in substrate binding (Emptage et al., 1983; Robbins and Stout, 1989a,b). Henceforth, it is assumed that, when a free radical reacts with the Fe/S cluster protein/enzyme, it, perhaps, sequesters out this free iron from the cluster that subsequently converts the active [4Fe-4S] protein to an inactive [3Fe-4S] cluster protein. In our model, the introduction of ONOO^−^ and ${\text{O}}_{2}^{•-}$ ions has caused increased production of inactive [3Fe-4S] clusters from active aconitase[4Fe-4S] and fumarase[4Fe-4S] enzymes by releasing Fe^3+^ into the system. This Fe^3+^ ion directly increased the rate of Fenton reaction resulting in enhanced production of Fe^2+^, ^•^OH and OH^−^. It was observed that the formation of ISCU [2Fe-2S] was possibly increased due to the higher level of accumulation of Fe^2+^ released from fenton reaction (Figure 5C).

**Figure 6:**
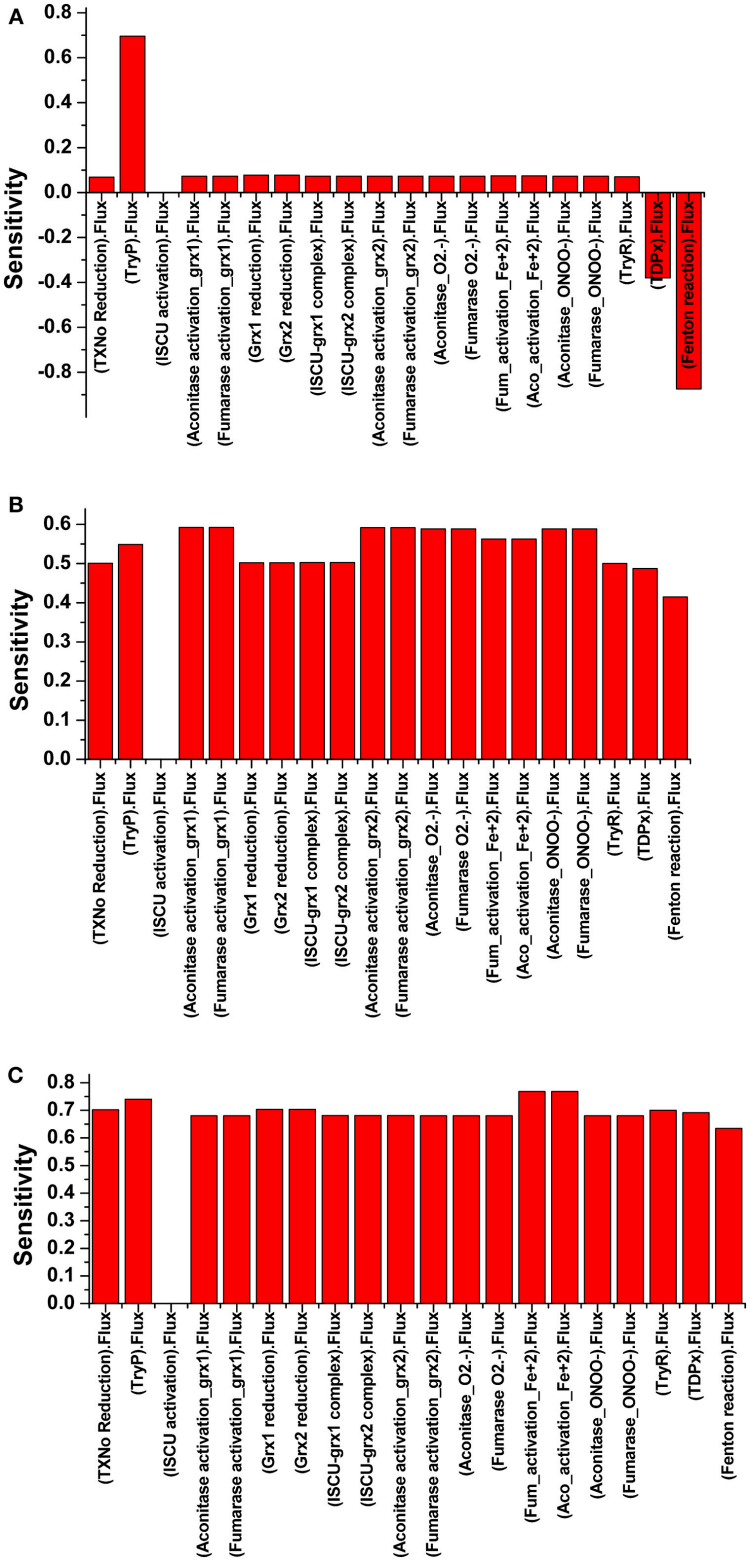
Illustration of Sensitivity analysis of **(A)** Model 2; **(B)** Model 3; and **(C)** Model 4. For sensitivity analyses in three models, Vmax_TryR_ was used to see its effect on the flux of other reactions. Analyses shows that there was nil effect on ISCU activation **(A)**, in contrast to the perturbed models with TryR reaction inhibition where all reactions except ISCU activation were found to be highly sensitive **(B,C)**.

The robustness i.e., the ability to cope with the kinetic perturbations to maintain the performance, of the kinetic model was confirmed by varying the values of kinetic parameters of the TryR reaction (Vmax_T[SH]2_, Km_T[S]2_ and Km_NADPH)_ (Table 3). Our results showed negligible variation in the concentration of T[SH]_2_ suggesting the robust nature of the model. A similar result in terms of robustness was obtained previously in the kinetic modeling study in *Trypanosoma cruzi* (Olin-Sandoval et al., 2012). To see the effect of TryR inhibition introduced in the basal kinetic model with radicals (M2), a sensitivity analysis of the pathways was performed. For this purpose, the TryR reaction parameter Vmax was selected to observe its influence on the reaction flux of all other reactions and a comparison was made among the three models (M2, M3, and M4). In all models, only ISCU (Iron sulfur cluster unit) activation was not affected by TryR reaction. It was observed that, the perturbed models with TryR reaction inhibition were highly sensitive to the reaction rate of TryR (Figure 6). Two downstream reactions, TDPx (neutralization of H_2_O_2_) and fenton reaction were found to be greatly sensitive to the rate of TryR reaction (Figures 6B,C). Although all reaction fluxes were found to be sensitive to T[SH]_2_ reduction rate including its own reaction flux, a 5 to 6-fold increase in sensitivity score was noted in all cases. Reactions related to Fe/S proteins activation, aconitase[4Fe-4S] and fumarase[4Fe-4S] via reduced Grx1/2 as well as their inactivation via free radicals were influenced at a greater rate suggesting direct influence of TryR on downstream reactions. These observations suggest that Fe/S proteins are directly connected to T[SH]_2_ level and a slight change in this would lead to the reduced level of activated Grx1/2, thereby, the activation of Fe/S proteins would be disturbed. Therefore, T[SH]_2_ emerged out as the most critical component of the redox metabolism and Fe/S cluster protein homeostasis in leishmania. The kinetic model built by our group is submitted to BioModel database (MODEL1811300001; Data Sheet 1) Chelliah et al. (2015).

**Table 3:** Robustness of the basal kinetic model (Model 1).

**Vmax T[SH]_**2**_**	**Folds**	**[T[SH]2]**
0.333	0.1 ×	49.2
3.33	1 ×	50
33.3	10 ×	50
**Km TS**_**2**_		
5	0.1 ×	50
50	1 ×	49.9
500	10 ×	49.3
**Km NADPH**		
2	0.1 ×	50
20	1 ×	50
200	10 ×	49.9

## Conclusion

In a nutshell, our kinetic models have demonstrated the importance of T[SH]_2_ in leishmanial cellular redox metabolism. Introducing the perturbation at the TryR reaction, our analyses suggests that the inhibition of TryR enzyme might be an important check point for disturbing the parasite's survival inside the host macrophages. By designing novel potent inhibitors against the TryR enzyme, inhibition of T[SH]_2_ reduction and thereby, the perturbation of activation and regulation of ISC proteins can be achieved. However, to prove this hypothesis, present kinetic models needs to be refined in order to reproduce longer oxidative stress conditions. Moreover, proper inhibition reactions should be incorporated in order to check which inhibition conditions suit the best to perturb the whole downstream metabolites production. This will require determination of experimental parameters solely in case of *Leishmania major*.

## Author Contributions

AK, NC, and SS contributed in the planning and experimentation of the study. AK and NC contributed in kinetic modeling. AK, NC, and SS participated in analyzing the results and writing of the manuscript.

### Conflict of Interest Statement

The authors declare that the research was conducted in the absence of any commercial or financial relationships that could be construed as a potential conflict of interest.

## Abbreviations

Arg: Arginine
Met: Methionine
Glu: Glutamate
Cys: Cysteine
Orn: Ornithine
Put: Putrescine
SAM: S-adenosylmethionine
dcSAM: S-adenosylmethioninamine
γEC: γ-L-glutamyl-L-cysteine
Spd: Spermidine
Gly: Glycine
GSH: Glutathione
T[SH]_2_: Trypanothione
Gspd: Glutathionylspermidine
TS_2_: Trypanothione disulfide
TXNo/r: Tryparedoxin oxidized/reduced
Grx: Glutaredoxin
ISCU: Iron sulfur cluster unit
Aco: Aconitase
Fum: Fumarase
SAMSyn: S-adnosylmethionine Synthase
SAMDc: S-adnosylmethionine decarboxylase
γECS: γEC synthetase
GS: Glutathione synthetase
TryS: Trypanothione synthetase
TryR: Trypanothione reductase
TryP: Trypaoredoxin peroxidase
ODC: Ornithine decarboxylase
SpdS: Spermidine synthase
TDPx: Glutathione peroxidase-like tryparedoxine
NADP^+^: Nicotinamide adenine dinucleotide phosphate
ISC: Iron sulfur cluster
CIA: Cytosolic iron sulfur protein assembly
GR: Glutathione reductase.
