# Supplementary material for: Understanding the Cross-Talk of Redox Metabolism and Fe-S Cluster Biogenesis in Leishmania Through Systems Biology Approach
Source: Front Cell Infect Microbiol. 2019 Feb 4;9:15. doi: 10.3389/fcimb.2019.00015 (PMC6369582; doi:10.3389/fcimb.2019.00015)
Supplement: Supplementary file 1 [file Table_1.DOCX]

**Table S1** Kinetic parameter values assigned to each reaction used in the models

| **SN** | **Reaction name** | **Km (μM)** | **Vmax**  **(μM. s^-1^)** | **S*_halve_*** | **n** | **kd (μM)** | **k1**  **(μM. s^-1^)** | **Kcat**  **(s^-1^)** | **Ka  (μM. s^-1^)** | **Kb  (μM.s^-1^)** | **References** |
| --- | --- | --- | --- | --- | --- | --- | --- | --- | --- | --- | --- |
| 1 | SAMsyn | - | 3.40E-03 | 250 | 2.3 |  |  |  |  |  | Pérez-Pertejo et al, 2003 |
| 2 | SAMdc | 3.80E+03 | 5.20E-03 |  |  |  |  |  |  |  | Willert et al, 2007 |
| 3 | Arginase | 1.25E+04 | 2.40E+03 |  |  |  |  |  |  |  | da Silva et al, 2008 |
| 4 | ODC | 4.20E+02 | 2.53E+04 |  |  |  |  |  |  |  | Osterman et al. 1994 |
| 5 | SpdS | 0.09 (dcSAM) | 1.98E-07 |  |  | 1.00E+05 |  |  |  |  | Taylor et al, 2008 |
|  |  | 205 (Put) |  |  |  |  |  |  |  |  |  |
| 6 | yECS | 9200 (Cys) | 1.8 |  |  | 93.7 |  |  |  |  | Agnihotri et al, 2016 |
|  |  | 1700 (Glu) |  |  |  |  |  |  |  |  |  |
| 7 | GS | 40 (GluCys) | 34 |  |  | 80 |  |  |  |  | Olin‐Sandoval et al, 2012 |
|  |  | 1200 (Gly) |  |  |  |  |  |  |  |  |  |
| 8 | TryS | 940 (Spd) | 80 |  |  |  |  |  |  |  | Oza et al, 2005 |
|  |  | 89 (GSH) |  |  |  |  |  |  |  |  |  |
| 9 | Try2 | 40 (TS2) | 80 |  |  |  |  |  |  |  | Oza et al, 2005 |
| 10 | TryR | 50 (TS2) | 3.33 |  |  |  |  |  |  |  | Mittal et al, 2005 |
|  |  | 20 (NADPH) |  |  |  |  |  |  |  |  |  |
| 11 | TXNo Reduction |  |  |  |  |  | 200 |  |  |  | Lüdemann et al, 1998 |
| 12 | TDPx |  |  |  |  |  |  | 15.4 | 193 | 2.2 | König et al, 2007 |
| 13 | TryP |  |  |  |  |  |  | 8.8 | 6.3 | 4.9 | König et al, 2007 |
| 14 | ISCU activation | 36.12 (Fe2+) | 1.11E+04 |  |  |  |  |  |  |  | Singh et al, 2018  Saas et al, 2000 |
|  |  | 1 (ISCU[2S]) |  |  |  |  |  |  |  |  |  |
| 15 | Aco_activation_grx1 | 2900 (Aconitase) | 1.77E+05 |  |  |  |  |  |  |  | Unciuleac  et al, 2007 |
|  |  | 36.12 ( ISC-Grx1r[4Fe-4S) |  |  |  |  |  |  |  |  |  |
| 16 | Fum_activation_grx1 | 2500 (fumarase) | 1.77E+05 |  |  |  |  |  |  |  | Unciuleac  et al, 2007 |
|  |  | 36.12 (ISC-Grx1r[4Fe-4S]) |  |  |  |  |  |  |  |  |  |
| 17 | Grx1 reduction |  |  |  |  |  | 2.83E+04 |  |  |  | Ceylan et al, 2010 |
| 18 | Grx2 reduction |  |  |  |  |  | 1.83E+04 |  |  |  | Ceylan et al, 2010 |
| 19 | ISCU-grx1 complex |  |  |  |  |  | 3.33E+05 |  |  |  | Shakamuri et al, 2012 |
| 20 | ISCU-grx2 complex |  |  |  |  |  | 3.33E+05 |  |  |  | Shakamuri et al, 2012 |
| 21 | Aco_activation_grx2 | 2900 (aconitase) | 1.77E+05 |  |  |  |  |  |  |  | Unciuleac et al, 2007 |
|  |  | 36.12 (ISC-Grx2r[4Fe-4S]) |  |  |  |  |  |  |  |  |  |
| 22 | Fum_activation_grx2 | 2900 (fumarase) | 1.77E+05 |  |  |  |  |  |  |  |  |
|  |  | 36.12 (ISC-Grx2r[4Fe-4S] |  |  |  |  |  |  |  |  |  |
| 23 | Aconitase_O_2_^●-^ |  |  |  |  |  | 3.5 |  |  |  | Crack et al, 2014  Castro et al, 1994 |
| 24 | Fumarase_O_2_^●-^ |  |  |  |  |  | 3.5 |  |  |  | Crack et al, 2014  Castro et al, 1994 |
| 25 | Fenton reaction |  |  |  |  |  | 7.60E+05 |  |  |  | Mittra et al, 2013 |
| 26 | Fum_activation_Fe^+2^ |  |  |  |  |  | 1.70E+03 |  |  |  | Unciuleac et al, 2007 |
| 27 | Aco_activation_Fe^+2^ |  |  |  |  |  | 1.70E+03 |  |  |  |  |
| 28 | Aco_ONOO- |  |  |  |  |  | 1.40E+14 |  |  |  | Castro et al, 1994 |
| 29 | Fum_ONOO- |  |  |  |  |  | 1.40E+14 |  |  |  |  |

**References**

Pérez‐Pertejo, Y., Reguera, R.M., Villa, H., García‐Estrada, C., Balaña‐Fouce, R., Pajares, M.A. and Ordóñez, D., (2003). Leishmania donovani methionine adenosyltransferase: Role of cysteine residues in the recombinant enzyme. Eur J Biochem, *270*(1) pp.28-35.

Willert, E.K., Fitzpatrick, R. and Phillips, M.A., (2007). Allosteric regulation of an essential trypanosome polyamine biosynthetic enzyme by a catalytically dead homolog. Proc Natl Acad Sci U S A. 104(20),8275-80.

da Silva, E.R., da Silva, M.F.L., Fischer, H., Mortara, R.A., Mayer, M.G., Framesqui, K., Silber, A.M. and Floeter-Winter, L.M., (2008). Biochemical and biophysical properties of a highly active recombinant arginase from Leishmania (Leishmania) amazonensis and subcellular localization of native enzyme. Mol Biochem Parasitol. 159(2),104-11. doi: 10.1016/j.molbiopara.2008.02.011.

Osterman, A., Grishin, N.V., Kinch, L.N. and Phillips, M.A., (1994). Formation of functional cross-species heterodimers of ornithine decarboxylase. Biochemistry. 33(46),13662-7.

Castro, L., Rodriguez, M. and Radi, R., (1994). Aconitase is readily inactivated by peroxynitrite, but not by its precursor, nitric oxide. J Biol Chem. 269(47),29409-15.

Agnihotri, P., Singh, S.P., Shakya, A.K. and Pratap, J.V., (2016). Biochemical and biophysical characterization of Leishmania donovani gamma-glutamylcysteine synthetase. Biochem Biophys Rep, *8*,127-138.

Olin‐Sandoval, V., González‐Chávez, Z., Berzunza‐Cruz, M., Martínez, I., Jasso‐Chávez, R., Becker, I., Espinoza, B., Moreno‐Sánchez, R. and Saavedra, E., (2012). Drug target validation of the trypanothione pathway enzymes through metabolic modelling. FEBS J, 279(10), 1811-1833.

Ceylan, S., Seidel, V., Ziebart, N., Berndt, C., Dirdjaja, N. and Krauth-Siegel, R.L., (2010). The dithiol glutaredoxins of African trypanosomes have distinct roles and are closely linked to the unique trypanothione metabolism. J Biol Chem. 285(45),35224-37. doi: 10.1074/jbc.M110.165860.

Mittal, M.K., Misra, S., Owais, M. and Goyal, N., (2005). Expression, purification, and characterization of Leishmania donovani trypanothione reductase in Escherichia coli. Protein Expr Purif. 40(2),279-86.

Cunningham, M.L. and Fairlamb, A.H., (1995). Trypanothione reductase from Leishmania donovani: purification, characterisation and inhibition by trivalent antimonials. Eur J Biochem. 230(2),460-8.

Feliciano, P.R., Gupta, S., Dyszy, F., Dias-Baruffi, M., Costa-Filho, A.J., Michels, P.A. and Nonato, M.C., (2012). Fumarate hydratase isoforms of Leishmania major: subcellular localization, structural and kinetic properties. Int J Biol Macromol. 51(1-2),25-31. doi: 10.1016/j.ijbiomac.2012.04.025.

Leroux, A.E., Maugeri, D.A., Cazzulo, J.J. and Nowicki, C., (2011). Functional characterization of NADP-dependent isocitrate dehydrogenase isozymes from Trypanosoma cruzi. Mol Biochem Parasitol. 177(1),61-4. doi: 10.1016/j.molbiopara.2011.01.010.

Mukherjee, A., Roy, G., Guimond, C. and Ouellette, M., (2009). The γ‐glutamylcysteine synthetase gene of Leishmania is essential and involved in response to oxidants Mol Microbiol. 74(4),914-27. Doi,10.1111/j.1365-2958.2009.06907.x.

Oza, S.L., Shaw, M.P., Wyllie, S. and Fairlamb, A.H., (2005). Trypanothione biosynthesis in Leishmania major. Mol Biochem Parasitol. 139(1),107-16.

Saas, J., Ziegelbauer, K., von Haeseler, A., Fast, B. and Boshart, M., (2000). A developmentally regulated aconitase related to iron-regulatory protein-1 is localized in the cytoplasm and in the mitochondrion of Trypanosoma brucei. J Biol Chem. 275(4),2745-55.

Shakamuri, P., Zhang, B. and Johnson, M.K., (2012). Monothiol glutaredoxins function in storing and transporting [Fe2S2] clusters assembled on IscU scaffold proteins. J Am Chem Soc 134(37),15213-6.

Singh, K.P., Anwar, S., Zaidi, A., Singh, K., Das, P., Gourinath, S. and Ali, V., (2018). LdIscU is a [2Fe-2S] scaffold protein which interacts with LdIscS and its expression is modulated by Fe-S proteins in Leishmania donovani. Int J Biol Macromol. 116,1128-1145. doi: 10.1016/j.ijbiomac.2018.05.060.

Crack, J.C., Stapleton, M.R., Green, J., Thomson, A.J. and Le Brun, N.E., (2014). Influence of association state and DNA binding on the O2-reactivity of [4Fe-4S] fumarate and nitrate reduction (FNR) regulator. Biochem J, 463(1), pp.83-92.

Taylor, M.C., Kaur, H., Blessington, B., Kelly, J.M. and Wilkinson, S.R., (2008). Validation of spermidine synthase as a drug target in African trypanosomes. Biochem J. 409(2),563-9.

Unciuleac, M.C., Chandramouli, K., Naik, S., Mayer, S., Huynh, B.H., Johnson, M.K. and Dean, D.R., (2007). In Vitro Activation of Apo-Aconitase Using a [4Fe-4S] Cluster-Loaded Form of the IscU [Fe− S] Cluster Scaffolding Protein. Biochemistry. 46(23),6812-21.

Lüdemann, H., Dormeyer, M., Sticherling, C., Stallmann, D., Follmann, H. and Krauth-Siegel, R.L., (1998). Trypanosoma brucei tryparedoxin, a thioredoxin‐like protein in African trypanosomes. FEBS Lett, 431(3), .381-385.

König, J. and Fairlamb, A.H., (2007). A comparative study of type I and type II tryparedoxin peroxidases in Leishmania major. FEBS J, 274(21), 5643-5658.

Mittra, B., Cortez, M., Haydock, A., Ramasamy, G., Myler, P.J. and Andrews, N.W.,( 2013). Iron uptake controls the generation of Leishmania infective forms through regulation of ROS levels. J Exp Med, 210(2), 401-416.
