# Supplementary material for: Understanding the Cross-Talk of Redox Metabolism and Fe-S Cluster Biogenesis in Leishmania Through Systems Biology Approach
Source: Front Cell Infect Microbiol. 2019 Feb 4;9:15. doi: 10.3389/fcimb.2019.00015 (PMC6369582; doi:10.3389/fcimb.2019.00015)
Supplement: Supplementary file 2 [file Table_2.DOCX]

**Table S2** Initial concentrations assigned to various components in reactions

| **Reaction component** | **Initial concentration (μM)** | **Reaction component** | **Initial concentration (μM)** |
| --- | --- | --- | --- |
| Met | 10 | Fe2+ | 0.0088 |
| Arg | 2000 | ISCU[2S] | 20 |
| Orn | 0 | ISCU[2Fe-2S] | 0 |
| dcSAM | 0 | Grx1 | 11.9 |
| Put | 0 | Grx2 | 13.8 |
| Cys | 10 | Grx1r | 0 |
| Glu | 10 | Grx2r | 0 |
| GluCys | 0 | aconitase | 6.7 |
| Gly | 10 | fumarase | 6.7 |
| Spd | 0 | aconitase[4Fe-4S] | 0 |
| GSH | 0 | fumarase[4Fe-4S] | 0 |
| Gspd | 0 | ISC-Grx2r[4Fe-4S] | 0 |
| TS2 | 50 | ISC-Grx1r[4Fe-4S] | 0 |
| NADPH | 80 | fumarase[3Fe-4S]^+^ | 0 |
| NADP | 0 | aconitase[3Fe-4S]^+^ | 0 |
| T[SH]2 | 0 | O_2_^●-^ | 100 |
| TXNo | 0 | H2O2 | 0 |
| TDPx | 0.2 | Fe+3 | 0 |
| H2O2 | 300 | ONOO- | 200 |
| TXNr | 28 |  |  |
