# Supplementary material for: Understanding the Cross-Talk of Redox Metabolism and Fe-S Cluster Biogenesis in Leishmania Through Systems Biology Approach
Source: Front Cell Infect Microbiol. 2019 Feb 4;9:15. doi: 10.3389/fcimb.2019.00015 (PMC6369582; doi:10.3389/fcimb.2019.00015)
Supplement: Supplementary file 3 [file Table_3.pdf]

Table S3. Ordinary Differential Equations for each of the kinetic reaction used in four models.

$$\frac{d([Met]\cdot V_{cell})}{dt} = -V_{cell}\cdot\left(\frac{V_{(SAMsyn)}\cdot[Met]^{h_{(SAMsyn)}}}{Shalve_{(SAMsyn)}{}^{h_{(SAMsyn)}}+[Met]^{h_{(SAMsyn)}}}\right)$$

$$\frac{d([SAM]\cdot V_{cell})}{dt} = +V_{cell}\cdot\left(\frac{V_{(SAMsyn)}\cdot[Met]^{h_{(SAMsyn)}}}{Shalve_{(SAMsyn)}{}^{h_{(SAMsyn)}}+[Met]^{h_{(SAMsyn)}}}\right) - V_{cell}\cdot\left(\frac{V_{(SAMdc)}\cdot[SAM]}{Km_{(SAMdc)}+[SAM]}\right)$$

$$\frac{d([dcSAM]\cdot V_{cell})}{dt} = +V_{cell}\cdot\left(\frac{V_{(SAMdc)}\cdot[SAM]}{Km_{(SAMdc)}+[SAM]}\right) - V_{cell}\cdot\left(\frac{VmPut_{(SpdS)}\cdot[Put]\cdot[dcSAM]}{KmdcSAM_{(SpdS)}\cdot[Put]+KmPut_{(SpdS)}\cdot[dcSAM]+[Put]\cdot[dcSAM]+KddcSAM_{(SpdS)}\cdot KmPut_{(SpdS)}}\right)$$

$$\frac{d([Arg]\cdot V_{cell})}{dt} = -V_{cell}\cdot\left(\frac{V_{(Arginase)}\cdot[Arg]}{Km_{(Arginase)}+[Arg]}\right)$$

$$\frac{d([Orn]\cdot V_{cell})}{dt} = +V_{cell}\cdot\left(\frac{V_{(Arginase)}\cdot[Arg]}{Km_{(Arginase)}+[Arg]}\right) - V_{cell}\cdot\left(\frac{V_{(ODC)}\cdot[Orn]}{Km_{(ODC)}+[Orn]}\right)$$

$$\frac{d([Put]\cdot V_{cell})}{dt} = +V_{cell}\cdot\left(\frac{V_{(ODC)}\cdot[Orn]}{Km_{(ODC)}+[Orn]}\right) - V_{cell}\cdot\left(\frac{VmPut_{(Spds)}\cdot[Put]\cdot[dcSAM]}{KmdcSAM_{(Spds)}\cdot[Put]+KmPut_{(Spds)}\cdot[dcSAM]+[Put]\cdot[dcSAM]+KddcSAM_{(Spds)}\cdot KmPut_{(Spds)}}\right)$$

$$\frac{d([Spd]\cdot V_{cell})}{dt} = +V_{cell}\cdot\left(\frac{VmPut_{(Spds)}\cdot[Put]\cdot[dcSAM]}{KmdcSAM_{(Spds)}\cdot[Put]+KmPut_{(Spds)}\cdot[dcSAM]+[Put]\cdot[dcSAM]+KddcSAM_{(Spds)}\cdot KmPut_{(Spds)}}\right) - V_{cell}\cdot\left(\frac{vmax_{(TryS1)}\cdot[Spd]\cdot[GSH]}{Kma_{(TryS1)}\cdot Kmb_{(TryS1)}+[Spd]\cdot Kmb_{(TryS1)}+[GSH]\cdot Kma_{(TryS1)}+[Spd]\cdot[GSH]}\right)$$

$$\frac{d([Cys]\cdot V_{cell})}{dt} = -V_{cell}\cdot\left(\frac{Vm_{(yECS)}\cdot[Cys]\cdot[Glu]}{Km2_{(yECS)}\cdot[Cys]+Km1_{(yECS)}\cdot[Glu]+[Cys]\cdot[Glu]+kd1_{(yECS)}\cdot Km2_{(yECS)}}\right)$$

$$\frac{d([Glu]\cdot V_{cell})}{dt} = -V_{cell}\cdot\left(\frac{Vm_{(yECS)}\cdot[Cys]\cdot[Glu]}{Km2_{(yECS)}\cdot[Cys]+Km1_{(yECS)}\cdot[Glu]+[Cys]\cdot[Glu]+kd1_{(yECS)}\cdot Km2_{(yECS)}}\right)$$

$$\frac{d([GluCys]\cdot V_{cell})}{dt} = +V_{cell}\cdot\left(\frac{Vm_{(yECS)}\cdot[Cys]\cdot[Glu]}{Km2_{(yECS)}\cdot[Cys]+Km1_{(yECS)}\cdot[Glu]+[Cys]\cdot[Glu]+kd1_{(yECS)}\cdot Km2_{(yECS)}}\right) - V_{cell}\cdot\left(\frac{Vm_{(GS)}\cdot[Gly]\cdot[GluCys]}{Km2_{(GS)}\cdot[Gly]+Km1_{(GS)}\cdot[GluCys]+[Gly]\cdot[GluCys]+kd1_{(GS)}\cdot Km2_{(GS)}}\right)$$

$$
\begin{aligned}
\frac{d([GSH]\cdot V_{cell})}{d\,t} = {} & - V_{cell}\cdot\left(\frac{Vm_{(GS)}\cdot[Gly]\cdot[GluCys]}{Km2_{(GS)}\cdot[Gly]+Km1_{(GS)}\cdot[GluCys]+[Gly]\cdot[GluCys]+kd1_{(GS)}\cdot Km2_{(GS)}}\right)\\
& + V_{cell}\cdot\left(\frac{Vm_{(GS)}\cdot[Gly]\cdot[GluCys]}{Km2_{(GS)}\cdot[Gly]+Km1_{(GS)}\cdot[GluCys]+[Gly]\cdot[GluCys]+kd1_{(GS)}\cdot Km2_{(GS)}}\right)\\
& - V_{cell}\cdot\left(\frac{vmax_{(TryS1)}\cdot[Spd]\cdot[GSH]}{Kma_{(TryS1)}\cdot Kmb_{(TryS1)}+[Spd]\cdot Kmb_{(TryS1)}+[GSH]\cdot Kma_{(TryS1)}+[Spd]\cdot[GSH]}\right)\\
& - V_{cell}\cdot\left(\frac{vmax_{(TryS2)}\cdot[Gspd]\cdot[GSH]}{Kma_{(TryS2)}\cdot Kmb_{(TryS2)}+[Gspd]\cdot Kmb_{(TryS2)}+[GSH]\cdot Kma_{(TryS2)}+[Gspd]\cdot[GSH]}\right)
\end{aligned}
$$

$$
\frac{d([Gly]\cdot V_{cell})}{d\,t} = - V_{cell}\cdot\left(\frac{Vm_{(GS)}\cdot[Gly]\cdot[GluCys]}{Km2_{(GS)}\cdot[Gly]+Km1_{(GS)}\cdot[GluCys]+[Gly]\cdot[GluCys]+kd1_{(GS)}\cdot Km2_{(GS)}}\right)
$$

$$
\begin{aligned}
\frac{d([Gspd]\cdot V_{cell})}{d\,t} = {} & + V_{cell}\cdot\left(\frac{vmax_{(TryS1)}\cdot[Spd]\cdot[GSH]}{Kma_{(TryS1)}\cdot Kmb_{(TryS1)}+[Spd]\cdot Kmb_{(TryS1)}+[GSH]\cdot Kma_{(TryS1)}+[Spd]\cdot[GSH]}\right)\\
& - V_{cell}\cdot\left(\frac{vmax_{(TryS2)}\cdot[Gspd]\cdot[GSH]}{Kma_{(TryS2)}\cdot Kmb_{(TryS2)}+[Gspd]\cdot Kmb_{(TryS2)}+[GSH]\cdot Kma_{(TryS2)}+[Gspd]\cdot[GSH]}\right)
\end{aligned}
$$

$$
\begin{aligned}
\frac{d([TS2]\cdot V_{cell})}{d\,t} = {} & + V_{cell}\cdot(k1_{(\text{"TXNo Reduction"})}\cdot[T[SH]2]\cdot[TXNo])\\
& + V_{cell}\cdot(k1_{(\text{"Grx1 reduction"})}\cdot[Grx1]\cdot[T[SH]2])\\
& + V_{cell}\cdot(k1_{(\text{"Grx2 reduction"})}\cdot[Grx2]\cdot[T[SH]2])\\
& - V_{cell}\cdot\left(\frac{V_{(TR)}\cdot[TS2]\cdot[NADPH]}{KmA_{(TR)}\cdot KmB_{(TR)}+KmB_{(TR)}\cdot[TS2]+KmA_{(TR)}\cdot[NADPH]}\right)
\end{aligned}
$$

$$
\begin{aligned}
\frac{d([T[SH]2]\cdot V_{cell})}{d\,t} = {} & - V_{cell}\cdot(k1_{(\text{"TXNo Reduction"})}\cdot[T[SH]2]\cdot[TXNo])\\
& - V_{cell}\cdot(k1_{(\text{"Grx1 reduction"})}\cdot[Grx1]\cdot[T[SH]2])\\
& - V_{cell}\cdot(k1_{(\text{"Grx2 reduction"})}\cdot[Grx2]\cdot[T[SH]2])\\
& + V_{cell}\cdot\left(\frac{vmax_{(TryS2)}\cdot[Gspd]\cdot[GSH]}{Kma_{(TryS2)}\cdot Kmb_{(TryS2)}+[Gspd]\cdot Kmb_{(TryS2)}+[GSH]\cdot Kma_{(TryS2)}+[Gspd]\cdot[GSH]}\right)\\
& + V_{cell}\cdot\left(\frac{V_{(TR)}\cdot[TS2]\cdot[NADPH]}{KmA_{(TR)}\cdot KmB_{(TR)}+KmB_{(TR)}\cdot[TS2]+KmA_{(TR)}\cdot[NADPH]}\right)
\end{aligned}
$$

$$
\frac{d([CO2]\cdot V_{cell})}{d\,t} = + V_{cell}\cdot\left(\frac{V_{(ODC)}\cdot[Orn]}{Km_{(ODC)}+[Orn]}\right)
$$

$$\frac{d([TXNo]\cdot V_{cell})}{d\,t} = -V_{cell}\cdot(k1_{(\text{"TXNo Reduction"})}\cdot[T[SH]2]\cdot[TXNo]) + V_{cell}\cdot\left(\frac{Kcat_{(TDPx)}\cdot[TDPx]\cdot[H2O2]\cdot[TXNr]}{Kb_{(TDPx)}\cdot[H2O2]+Ka_{(TDPx)}\cdot[TXNr]+[H2O2]\cdot[TXNr]}\right) + V_{cell}\cdot\left(\frac{Kcat_{(TryP)}\cdot[TryP]\cdot[H2O2]\cdot[TXNr]}{Kb_{(TryP)}\cdot[H2O2]+Ka_{(TryP)}\cdot[TXNr]+[H2O2]\cdot[TXNr]}\right)$$

$$\frac{d([TXNr]\cdot V_{cell})}{d\,t} = +V_{cell}\cdot(k1_{(\text{"TXNo Reduction"})}\cdot[T[SH]2]\cdot[TXNo]) - V_{cell}\cdot\left(\frac{Kcat_{(TDPx)}\cdot[TDPx]\cdot[H2O2]\cdot[TXNr]}{Kb_{(TDPx)}\cdot[H2O2]+Ka_{(TDPx)}\cdot[TXNr]+[H2O2]\cdot[TXNr]}\right) - V_{cell}\cdot\left(\frac{Kcat_{(TryP)}\cdot[TryP]\cdot[H2O2]\cdot[TXNr]}{Kb_{(TryP)}\cdot[H2O2]+Ka_{(TryP)}\cdot[TXNr]+[H2O2]\cdot[TXNr]}\right)$$

$$\frac{d([H2O]\cdot V_{cell})}{d\,t} = +V_{cell}\cdot\left(\frac{Kcat_{(TDPx)}\cdot[TDPx]\cdot[H2O2]\cdot[TXNr]}{Kb_{(TDPx)}\cdot[H2O2]+Ka_{(TDPx)}\cdot[TXNr]+[H2O2]\cdot[TXNr]}\right) + V_{cell}\cdot\left(\frac{Kcat_{(TryP)}\cdot[TryP]\cdot[H2O2]\cdot[TXNr]}{Kb_{(TryP)}\cdot[H2O2]+Ka_{(TryP)}\cdot[TXNr]+[H2O2]\cdot[TXNr]}\right)$$

$$\frac{d([H2O2]\cdot V_{cell})}{d\,t} = -V_{cell}\cdot\left(\frac{Kcat_{(TDPx)}\cdot[TDPx]\cdot[H2O2]\cdot[TXNr]}{Kb_{(TDPx)}\cdot[H2O2]+Ka_{(TDPx)}\cdot[TXNr]+[H2O2]\cdot[TXNr]}\right) - V_{cell}\cdot\left(\frac{Kcat_{(TryP)}\cdot[TryP]\cdot[H2O2]\cdot[TXNr]}{Kb_{(TryP)}\cdot[H2O2]+Ka_{(TryP)}\cdot[TXNr]+[H2O2]\cdot[TXNr]}\right) + V_{cell}\cdot(k1_{(\text{"Aconitase\_O2.-"})}\cdot[\text{"O2.-"}]\cdot[\text{"aconitase[4Fe-4S]"}]) + V_{cell}\cdot(k1_{(\text{"Fumarase O2.-"})}\cdot[\text{"O2.-"}]\cdot[\text{"fumarase[4Fe-4S]"}]) - V_{cell}\cdot(k1_{(\text{"Fenton reaction"})}\cdot[H2O2]\cdot[\text{"Fe+3"}])$$

$$\frac{d([aconitase]\cdot V_{cell})}{d\,t} = -V_{cell}\cdot\left(\frac{V_{(Aco\_activation\_grx1)}\cdot[aconitase]\cdot["ISC\text{-}Grx1r[4Fe\text{-}4S]"]}{KmA_{(Aco\_activation\_grx1)}\cdot KmB_{(Aco\_activation\_grx1)}+KmB_{(Aco\_activation\_grx1)}\cdot[aconitase]+KmA_{(Aco\_activation\_grx1)}\cdot["ISC\text{-}Grx1r[4Fe\text{-}4S]"]}\right)$$

$$-V_{cell}\cdot\left(\frac{V_{(Aco\_activation\_grx2)}\cdot[aconitase]\cdot["ISC\text{-}Grx2r[4Fe\text{-}4S]"]}{KmA_{(Aco\_activation\_grx2)}\cdot KmB_{(Aco\_activation\_grx2)}+KmB_{(Aco\_activation\_grx2)}\cdot[aconitase]+KmA_{(Aco\_activation\_grx2)}\cdot["ISC\text{-}Grx2r[4Fe\text{-}4S]"]}\right)$$

$$\frac{d(["fumarase[4Fe\text{-}4S]"]\cdot V_{cell})}{d\,t} = +V_{cell}\cdot\left(\frac{V_{(Fum\_activation\_grx1)}\cdot[Fumarase]\cdot["ISC\text{-}Grx1r[4Fe\text{-}4S]"]}{KmA_{(Fum\_activation\_grx1)}\cdot KmB_{(Fum\_activation\_grx1)}+KmB_{(Fum\_activation\_grx1)}\cdot[Fumarase]+KmA_{(Fum\_activation\_grx1)}\cdot["ISC\text{-}Grx1r[4Fe\text{-}4S]"]}\right)$$

$$+V_{cell}\cdot\left(\frac{V_{(Fum\_activation\_grx2)}\cdot[Fumarase]\cdot["ISC\text{-}Grx2r[4Fe\text{-}4S]"]}{KmA_{(Fum\_activation\_grx2)}\cdot KmB_{(Fum\_activation\_grx2)}+KmB_{(Fum\_activation\_grx2)}\cdot[Fumarase]+KmA_{(Fum\_activation\_grx2)}\cdot["ISC\text{-}Grx2r[4Fe\text{-}4S]"]}\right)$$

$$-V_{cell}\cdot(k1_{("Fumarase\ O2.-")}\cdot["O2.-"]\cdot["fumarase[4Fe\text{-}4S]"])$$

$$+V_{cell}\cdot(k1_{("Fum\_activation\_Fe+2")}\cdot["fumarase[3Fe\text{-}4S]+"]\cdot["Fe+2"])$$

$$-V_{cell}\cdot(k1_{("Fum\_ONOO-")}\cdot["fumarase[4Fe\text{-}4S]"]\cdot["ONOO-"])$$

$$\frac{d([Fumarase]\cdot V_{cell})}{d\,t} = -V_{cell}\cdot\left(\frac{V_{(Fum\_activation\_grx1)}\cdot[Fumarase]\cdot["ISC\text{-}Grx1r[4Fe\text{-}4S]"]}{KmA_{(Fum\_activation\_grx1)}\cdot KmB_{(Fum\_activation\_grx1)}+KmB_{(Fum\_activation\_grx1)}\cdot[Fumarase]+KmA_{(Fum\_activation\_grx1)}\cdot["ISC\text{-}Grx1r[4Fe\text{-}4S]"]}\right)$$

$$-V_{cell}\cdot\left(\frac{V_{(Fum\_activation\_grx2)}\cdot[Fumarase]\cdot["ISC\text{-}Grx2r[4Fe\text{-}4S]"]}{KmA_{(Fum\_activation\_grx2)}\cdot KmB_{(Fum\_activation\_grx2)}+KmB_{(Fum\_activation\_grx2)}\cdot[Fumarase]+KmA_{(Fum\_activation\_grx2)}\cdot["ISC\text{-}Grx2r[4Fe\text{-}4S]"]}\right)$$

$$\frac{d([Grx1]\cdot V_{cell})}{d\,t} = -V_{cell}\cdot(k1_{("Grx1\ reduction")}\cdot[Grx1]\cdot[T[SH]2])$$

$$\frac{d([Grx1r]\cdot V_{cell})}{d\,t} = +V_{cell}\cdot(k1_{("Grx1\ reduction")}\cdot[Grx1]\cdot[T[SH]2])$$

$$-V_{cell}\cdot(k1_{("ISCU\text{-}grx1\ complex")}\cdot["ISCU[2Fe\text{-}2S]"]\cdot[Grx1r])$$

$$\frac{d([Grx2]\cdot V_{cell})}{d\,t} = -V_{cell}\cdot(k1_{("Grx2\ reduction")}\cdot[Grx2]\cdot[T[SH]2])$$

$$\frac{d([Grx2]\cdot V_{cell})}{d\,t} = -V_{cell}\cdot\left(k1_{(\text{"Grx2 reduction"})}\cdot[Grx2]\cdot[T[SH]2]\right)$$

$$\frac{d([Grx2r]\cdot V_{cell})}{d\,t} = +V_{cell}\cdot\left(k1_{(\text{"Grx2 reduction"})}\cdot[Grx2]\cdot[T[SH]2]\right) - V_{cell}\cdot\left(k1_{(\text{"ISCU-grx2 complex"})}\cdot[\text{"ISCU[2Fe-2S]"}]\cdot[Grx2r]\right)$$

$$\frac{d([\text{"aconitase[4Fe-4S]"}]\cdot V_{cell})}{d\,t} = +V_{cell}\cdot\left(\frac{V_{(Aco\_activation\_grx1)}\cdot[aconitase]\cdot[\text{"ISC-Grx1r[4Fe-4S]"}]}{KmA_{(Aco\_activation\_grx1)}\cdot KmB_{(Aco\_activation\_grx1)}+KmB_{(Aco\_activation\_grx1)}\cdot[aconitase]+KmA_{(Aco\_activation\_grx1)}\cdot[\text{"ISC-Grx1r[4Fe-4S]"}]}\right)$$
$$+V_{cell}\cdot\left(\frac{V_{(Aco\_activation\_grx2)}\cdot[aconitase]\cdot[\text{"ISC-Grx2r[4Fe-4S]"}]}{KmA_{(Aco\_activation\_grx2)}\cdot KmB_{(Aco\_activation\_grx2)}+KmB_{(Aco\_activation\_grx2)}\cdot[aconitase]+KmA_{(Aco\_activation\_grx2)}\cdot[\text{"ISC-Grx2r[4Fe-4S]"}]}\right)$$
$$-V_{cell}\cdot\left(k1_{(\text{"Aconitase\_O2.-"})}\cdot[\text{"O2.-"}]\cdot[\text{"aconitase[4Fe-4S]"}]\right)$$
$$+V_{cell}\cdot\left(k1_{(\text{"Aco\_activation\_Fe+2"})}\cdot[\text{"aconitase[3Fe-4S]+"}]\cdot[\text{"Fe+2"}]\right)$$
$$-V_{cell}\cdot\left(k1_{(\text{"Aco\_ONOO-"})}\cdot[\text{"aconitase[4Fe-4S]"}]\cdot[\text{"ONOO-"}]\right)$$

$$\frac{d([ISCU]\cdot V_{cell})}{d\,t} = +V_{cell}\cdot\left(\frac{V_{(Aco\_activation\_grx1)}\cdot[aconitase]\cdot[\text{"ISC-Grx1r[4Fe-4S]"}]}{KmA_{(Aco\_activation\_grx1)}\cdot KmB_{(Aco\_activation\_grx1)}+KmB_{(Aco\_activation\_grx1)}\cdot[aconitase]+KmA_{(Aco\_activation\_grx1)}\cdot[\text{"ISC-Grx1r[4Fe-4S]"}]}\right)$$
$$+V_{cell}\cdot\left(\frac{V_{(Fum\_activation\_grx1)}\cdot[Fumarase]\cdot[\text{"ISC-Grx1r[4Fe-4S]"}]}{KmA_{(Fum\_activation\_grx1)}\cdot KmB_{(Fum\_activation\_grx1)}+KmB_{(Fum\_activation\_grx1)}\cdot[Fumarase]+KmA_{(Fum\_activation\_grx1)}\cdot[\text{"ISC-Grx1r[4Fe-4S]"}]}\right)$$
$$+V_{cell}\cdot\left(\frac{V_{(Aco\_activation\_grx2)}\cdot[aconitase]\cdot[\text{"ISC-Grx2r[4Fe-4S]"}]}{KmA_{(Aco\_activation\_grx2)}\cdot KmB_{(Aco\_activation\_grx2)}+KmB_{(Aco\_activation\_grx2)}\cdot[aconitase]+KmA_{(Aco\_activation\_grx2)}\cdot[\text{"ISC-Grx2r[4Fe-4S]"}]}\right)$$
$$+V_{cell}\cdot\left(\frac{V_{(Fum\_activation\_grx2)}\cdot[Fumarase]\cdot[\text{"ISC-Grx2r[4Fe-4S]"}]}{KmA_{(Fum\_activation\_grx2)}\cdot KmB_{(Fum\_activation\_grx2)}+KmB_{(Fum\_activation\_grx2)}\cdot[Fumarase]+KmA_{(Fum\_activation\_grx2)}\cdot[\text{"ISC-Grx2r[4Fe-4S]"}]}\right)$$

$$\frac{d(["ISCU[2Fe-2S]"] \cdot V_{cell})}{dt} = +V_{cell} \cdot \left(\frac{V_{("ISCU\ activation")} \cdot ["Fe+2"] \cdot ["Fe+2"]}{KmA_{("ISCU\ activation")} \cdot KmB_{("ISCU\ activation")} + KmB_{("ISCU\ activation")} \cdot ["Fe+2"] + KmA_{("ISCU\ activation")} \cdot ["Fe+2"]} \right)$$
$$- V_{cell} \cdot (k1_{("ISCU-grx1\ complex")} \cdot ["ISCU[2Fe-2S]"] \cdot [Grx1r])$$
$$- V_{cell} \cdot (k1_{("ISCU-grx2\ complex")} \cdot ["ISCU[2Fe-2S]"] \cdot [Grx2r])$$

$$\frac{d([ISCU[2S]] \cdot V_{cell})}{dt} = -V_{cell} \cdot \left(\frac{V_{("ISCU\ activation")} \cdot ["Fe+2"] \cdot ["Fe+2"]}{KmA_{("ISCU\ activation")} \cdot KmB_{("ISCU\ activation")} + KmB_{("ISCU\ activation")} \cdot ["Fe+2"] + KmA_{("ISCU\ activation")} \cdot ["Fe+2"]} \right)$$

$$\frac{d(["ISC-Grx1r[4Fe-4S]"] \cdot V_{cell})}{dt} = -V_{cell} \cdot \left(\frac{V_{(Aco\_activation\_grx1)} \cdot [aconitase] \cdot ["ISC-Grx1r[4Fe-4S]"]}{KmA_{(Aco\_activation\_grx1)} \cdot KmB_{(Aco\_activation\_grx1)} + KmB_{(Aco\_activation\_grx1)} \cdot [aconitase] + KmA_{(Aco\_activation\_grx1)} \cdot ["ISC-Grx1r[4Fe-4S]"]} \right)$$
$$- V_{cell} \cdot \left(\frac{V_{(Fum\_activation\_grx1)} \cdot [Fumarase] \cdot ["ISC-Grx1r[4Fe-4S]"]}{KmA_{(Fum\_activation\_grx1)} \cdot KmB_{(Fum\_activation\_grx1)} + KmB_{(Fum\_activation\_grx1)} \cdot [Fumarase] + KmA_{(Fum\_activation\_grx1)} \cdot ["ISC-Grx1r[4Fe-4S]"]} \right)$$
$$+ V_{cell} \cdot (k1_{("ISCU-grx1\ complex")} \cdot ["ISCU[2Fe-2S]"] \cdot [Grx1r])$$

$$\frac{d(["ISC-Grx2r[4Fe-4S]"] \cdot V_{cell})}{dt} = +V_{cell} \cdot (k1_{("ISCU-grx2\ complex")} \cdot ["ISCU[2Fe-2S]"] \cdot [Grx2r])$$
$$- V_{cell} \cdot \left(\frac{V_{(Aco\_activation\_grx2)} \cdot [aconitase] \cdot ["ISC-Grx2r[4Fe-4S]"]}{KmA_{(Aco\_activation\_grx2)} \cdot KmB_{(Aco\_activation\_grx2)} + KmB_{(Aco\_activation\_grx2)} \cdot [aconitase] + KmA_{(Aco\_activation\_grx2)} \cdot ["ISC-Grx2r[4Fe-4S]"]} \right)$$
$$- V_{cell} \cdot \left(\frac{V_{(Fum\_activation\_grx2)} \cdot [Fumarase] \cdot ["ISC-Grx2r[4Fe-4S]"]}{KmA_{(Fum\_activation\_grx2)} \cdot KmB_{(Fum\_activation\_grx2)} + KmB_{(Fum\_activation\_grx2)} \cdot [Fumarase] + KmA_{(Fum\_activation\_grx2)} \cdot ["ISC-Grx2r[4Fe-4S]"]} \right)$$

$$\frac{d(["O2.-"] \cdot V_{cell})}{dt} = -V_{cell} \cdot (k1_{("Aconitase\_O2.-")} \cdot ["O2.-"] \cdot ["aconitase[4Fe-4S]"])$$
$$- V_{cell} \cdot (k1_{("Fumarase\ O2.-")} \cdot ["O2.-"] \cdot ["fumarase[4Fe-4S]"])$$

$$\frac{d([.OH]\cdot V_{cell})}{d\,t} = +V_{cell}\cdot(k1_{(\text{"Fenton reaction"})}\cdot[H2O2]\cdot[\text{"Fe+3"}])$$

$$\frac{d([\text{"Fe+3"}]\cdot V_{cell})}{d\,t} = +V_{cell}\cdot(k1_{(\text{"Aconitase\_O2.-"})}\cdot[\text{"O2.-"}]\cdot[\text{"aconitase[4Fe-4S]"}])$$
$$+V_{cell}\cdot(k1_{(\text{"Fumarase O2.-"})}\cdot[\text{"O2.-"}]\cdot[\text{"fumarase[4Fe-4S]"}])$$
$$-V_{cell}\cdot(k1_{(\text{"Fenton reaction"})}\cdot[H2O2]\cdot[\text{"Fe+3"}])$$
$$+V_{cell}\cdot(k1_{(\text{"Aco\_ONOO-"})}\cdot[\text{"aconitase[4Fe-4S]"}]\cdot[\text{"ONOO-"}])$$
$$+V_{cell}\cdot(k1_{(\text{"Fum\_ONOO-"})}\cdot[\text{"fumarase[4Fe-4S]"}]\cdot[\text{"ONOO-"}])$$

$$\frac{d([\text{"OH-"}]\cdot V_{cell})}{d\,t} = +V_{cell}\cdot(k1_{(\text{"Fenton reaction"})}\cdot[H2O2]\cdot[\text{"Fe+3"}])$$

$$\frac{d([\text{"aconitase[3Fe-4S]+"}]\cdot V_{cell})}{d\,t} = +V_{cell}\cdot(k1_{(\text{"Aconitase\_O2.-"})}\cdot[\text{"O2.-"}]\cdot[\text{"aconitase[4Fe-4S]"}])$$
$$-V_{cell}\cdot(k1_{(\text{"Aco\_activation\_Fe+2"})}\cdot[\text{"aconitase[3Fe-4S]+"}]\cdot[\text{"Fe+2"}])$$
$$+V_{cell}\cdot(k1_{(\text{"Aco\_ONOO-"})}\cdot[\text{"aconitase[4Fe-4S]"}]\cdot[\text{"ONOO-"}])$$

$$\frac{d([\text{"fumarase[3Fe-4S]+"}]\cdot V_{cell})}{d\,t} = +V_{cell}\cdot(k1_{(\text{"Fumarase O2.-"})}\cdot[\text{"O2.-"}]\cdot[\text{"fumarase[4Fe-4S]"}])$$
$$-V_{cell}\cdot(k1_{(\text{"Fum\_activation\_Fe+2"})}\cdot[\text{"fumarase[3Fe-4S]+"}]\cdot[\text{"Fe+2"}])$$
$$+V_{cell}\cdot(k1_{(\text{"Fum\_ONOO-"})}\cdot[\text{"fumarase[4Fe-4S]"}]\cdot[\text{"ONOO-"}])$$

$$\frac{d([\text{"ONOO-"}]\cdot V_{cell})}{d\,t} = -V_{cell}\cdot(k1_{(\text{"Aco\_ONOO-"})}\cdot[\text{"aconitase[4Fe-4S]"}]\cdot[\text{"ONOO-"}])$$
$$-V_{cell}\cdot(k1_{(\text{"Fum\_ONOO-"})}\cdot[\text{"fumarase[4Fe-4S]"}]\cdot[\text{"ONOO-"}])$$

$$\frac{d([NADP]\cdot V_{cell})}{d\,t} = +V_{cell}\cdot\left(\frac{V_{(TR)}\cdot[TS2]\cdot[NADPH]}{KmA_{(TR)}\cdot KmB_{(TR)}+KmB_{(TR)}\cdot[TS2]+KmA_{(TR)}\cdot[NADPH]}\right)$$

$$\frac{d([NADPH]\cdot V_{cell})}{d\,t} = -V_{cell}\cdot\left(\frac{V_{(TR)}\cdot[TS2]\cdot[NADPH]}{KmA_{(TR)}\cdot KmB_{(TR)}+KmB_{(TR)}\cdot[TS2]+KmA_{(TR)}\cdot[NADPH]}\right)$$
