# Supplementary material for: Understanding the Cross-Talk of Redox Metabolism and Fe-S Cluster Biogenesis in Leishmania Through Systems Biology Approach
Source: Front Cell Infect Microbiol. 2019 Feb 4;9:15. doi: 10.3389/fcimb.2019.00015 (PMC6369582; doi:10.3389/fcimb.2019.00015)
Supplement: Supplementary file 4 [file Table_4.docx]

**Table S4.** Experimentally determined Ki values of TryR enzymes.

| **Sr No** | **Compound name** | **Trypanothione reductase Ki values (µM)** | **References** |
| --- | --- | --- | --- |
| 1. | Clomipramine | 6.5 | Werbovetz et al, 2000 |
|  | Chlorpromazine | 10-68 |  |
|  | Phenylpropyl spermine | 3.5 |  |
| 2. | Chlorhexidine | 6.13 ± 1.65 | Beig et al, 2015 |
|  | Mepacrine | 20.71 ± 5.47 |  |
|  | BG237 | 42.13 ± 4.38 |  |
| 3. | TNQ­2 | 2.5-5 | Zani et al, 2003 |
| 4. | RDS777 | 0.25 ± 0.18 | Saccoliti et al, 2017 |

Beig, M., Oellien, F., Garoff, L., Noack, S., Krauth-Siegel, R.L. and Selzer, P.M., (2015). Trypanothione reductase: a target protein for a combined in vitro and in silico screening approach. PLoS Negl Trop Dis, 9(6), e0003773, doi: 10.1371/journal.pntd.0003773.

Saccoliti, F., Angiulli, G., Pupo, G., Pescatori, L., Madia, V.N., Messore, A., Colotti, G., Fiorillo, A., Scipione, L., Gramiccia, M. and Di Muccio, T., 2017. Inhibition of Leishmania infantum trypanothione reductase by diaryl sulfide derivatives. J Enzyme Inhib Med Chem, 32(1), 304-310.

Werbovetz, K.A., (2000). Target-based drug discovery for malaria, leishmaniasis, and trypanosomiasis. Curr Med Chem, 7(8), 835-860.

Zani, C.L. and Fairlamb, A.H., (2003). 8-Methoxy-naphtho [2, 3-b] thiophen-4, 9-quinone, a non-competitive inhibitor of trypanothione reductase. Mem Inst Oswaldo Cruz, 98(4), 565-568.
