# Supplementary material for: Understanding the Cross-Talk of Redox Metabolism and Fe-S Cluster Biogenesis in Leishmania Through Systems Biology Approach
Source: Front Cell Infect Microbiol. 2019 Feb 4;9:15. doi: 10.3389/fcimb.2019.00015 (PMC6369582; doi:10.3389/fcimb.2019.00015)
Supplement: Supplementary file 5 [file Data_Sheet_1.pdf]

Data Sheet 1: Representation of Basal Kinetic model in detail as SBML file format.


```
<?xml version='1.0' encoding='UTF-8' standalone='no'?>
<sbml xmlns="http://www.sbml.org/sbml/level2/version4" level="2"
version="4">
  <model id="New_Model_1" metaid="COPASI0" name="Mathematical
model of FeS clusters and redox metabolism in Leishmania">
    <notes>
      <body xmlns="http://www.w3.org/1999/xhtml">
     <p>Fe/S clusters are versatile cofactors of various proteins
and carried out much broad range of essential biological
processes viz., TCA cycle, redox homeostasis, etc. Although,
several Fe/S cluster proteins and their roles have been
identified in Leishmania, some of the components of how T[SH]2 is
involved in the regulation of Fe/S proteins yet to be explored.
In order to understand the same, a systems biology approach was
adopted to get an insight of the overall picture to unravel how
T[SH]2 synthesis and reduction is linked with the regulation of
Fe/S cluster proteins and controls the redox homeostasis at
larger scale.</p>
  </body>
    </notes>
    <annotation>
      <COPASI xmlns="http://www.copasi.org/static/sbml">
        <rdf:RDF xmlns:dcterms="http://purl.org/dc/terms/"
xmlns:rdf="http://www.w3.org/1999/02/22-rdf-syntax-ns#">
          <rdf:Description rdf:about="#COPASI0">
            <dcterms:created>
              <rdf:Description>

<dcterms:W3CDTF>2017-06-28T16:13:30Z</dcterms:W3CDTF>
              </rdf:Description>
            </dcterms:created>
          </rdf:Description>
        </rdf:RDF>
      </COPASI>
    </annotation>
      <listOfFunctionDefinitions>
      <functionDefinition id="Bi__irreversible" metaid="COPASI32"
name="Bi (irreversible)" sboTerm="SBO:0000151">
        <notes>
          <body xmlns="http://www.w3.org/1999/xhtml">
            <pre>enzymatic rate law for irreversible non-
modulated non-interacting bireactant enzymes</pre>
          </body>
        </notes>
        <annotation>
          <rdf:RDF xmlns:rdf="http://www.w3.org/1999/02/22-rdf-
syntax-ns#" xmlns:dc="http://purl.org/dc/elements/1.1/"
xmlns:dcterms="http://purl.org/dc/terms/"
xmlns:vCard="http://www.w3.org/2001/vcard-rdf/3.0#"
xmlns:bqbiol="http://biomodels.net/biology-qualifiers/"
xmlns:bqmodel="http://biomodels.net/model-qualifiers/">
            <rdf:Description rdf:about="#COPASI32">
```

```
      <bqbiol:is>
      <rdf:Bag>
      <rdf:li
rdf:resource="http://identifiers.org/sbo/SBO:0000151"/>
      </rdf:Bag>
      </bqbiol:is>
      </rdf:Description>

          </rdf:RDF>
          <COPASI xmlns="http://www.copasi.org/static/sbml">
            <rdf:RDF
xmlns:CopasiMT="http://www.copasi.org/RDF/MiriamTerms#"
xmlns:dcterms="http://purl.org/dc/terms/"
xmlns:rdf="http://www.w3.org/1999/02/22-rdf-syntax-ns#">
              <rdf:Description rdf:about="#COPASI32">
                <dcterms:created>
                  <rdf:Description>

<dcterms:W3CDTF>2018-01-04T11:04:02Z</dcterms:W3CDTF>
                  </rdf:Description>
                </dcterms:created>
                <CopasiMT:is
rdf:resource="urn:miriam:obo.sbo:SBO:0000151"/>
              </rdf:Description>
            </rdf:RDF>
          </COPASI>
        </annotation>
        <math xmlns="http://www.w3.org/1998/Math/MathML">
          <lambda>
            <bvar>
              <ci> vmax </ci>
            </bvar>
            <bvar>
              <ci> A </ci>
            </bvar>
            <bvar>
              <ci> B </ci>
            </bvar>
            <bvar>
              <ci> Kma </ci>
            </bvar>
            <bvar>
              <ci> Kmb </ci>
            </bvar>
            <apply>
              <divide/>
              <apply>
                <times/>
                <ci> vmax </ci>
                <ci> A </ci>
                <ci> B </ci>
              </apply>
```

```
              <apply>
                <plus/>
                <apply>
                  <times/>
                  <ci> Kma </ci>
                  <ci> Kmb </ci>
                </apply>
                <apply>
                  <times/>
                  <ci> A </ci>
                  <ci> Kmb </ci>
                </apply>
                <apply>
                  <times/>
                  <ci> B </ci>
                  <ci> Kma </ci>
                </apply>
                <apply>
                  <times/>
                  <ci> A </ci>
                  <ci> B </ci>
                </apply>
              </apply>
            </apply>
          </lambda>
        </math>
            </functionDefinition>
      <functionDefinition id="SpdSynth_1" metaid="COPASI33"
name="SpdSynth_1">
        <annotation>
          <COPASI xmlns="http://www.copasi.org/static/sbml">
            <rdf:RDF xmlns:dcterms="http://purl.org/dc/terms/"
xmlns:rdf="http://www.w3.org/1999/02/22-rdf-syntax-ns#">
              <rdf:Description rdf:about="#COPASI33">
                <dcterms:created>
                  <rdf:Description>

<dcterms:W3CDTF>2017-07-03T09:54:10Z</dcterms:W3CDTF>
                  </rdf:Description>
                </dcterms:created>
              </rdf:Description>
            </rdf:RDF>
          </COPASI>
        </annotation>
        <math xmlns="http://www.w3.org/1998/Math/MathML">
          <lambda>
            <bvar>
              <ci> KddcSAM </ci>
            </bvar>
            <bvar>
              <ci> KmPut </ci>
            </bvar>
```

```
            <bvar>
              <ci> KmdcSAM </ci>
            </bvar>
            <bvar>
              <ci> Put </ci>
            </bvar>
            <bvar>
              <ci> VmPut </ci>
            </bvar>
            <bvar>
              <ci> dcSAM </ci>
            </bvar>
            <apply>
              <divide/>
              <apply>
                <times/>
                <ci> VmPut </ci>
                <ci> Put </ci>
                <ci> dcSAM </ci>
              </apply>
              <apply>
                <plus/>
                <apply>
                  <times/>
                  <ci> KmdcSAM </ci>
                  <ci> Put </ci>
                </apply>
                <apply>
                  <times/>
                  <ci> KmPut </ci>
                  <ci> dcSAM </ci>
                </apply>
                <apply>
                  <times/>
                  <ci> Put </ci>
                  <ci> dcSAM </ci>
                </apply>
                <apply>
                  <times/>
                  <ci> KddcSAM </ci>
                  <ci> KmPut </ci>
                </apply>
              </apply>
            </apply>
          </lambda>
        </math>
            </functionDefinition>
      <functionDefinition
id="Henri_Michaelis_Menten__irreversible__3" metaid="COPASI34"
name="Henri-Michaelis-Menten (irreversible)_3">
        <annotation>
          <COPASI xmlns="http://www.copasi.org/static/sbml">
```

```
            <rdf:RDF xmlns:dcterms="http://purl.org/dc/terms/"
xmlns:rdf="http://www.w3.org/1999/02/22-rdf-syntax-ns#">
              <rdf:Description rdf:about="#COPASI34">
                <dcterms:created>
                  <rdf:Description>

<dcterms:W3CDTF>2018-03-08T12:34:15Z</dcterms:W3CDTF>
                  </rdf:Description>
                </dcterms:created>
              </rdf:Description>
            </rdf:RDF>
          </COPASI>
        </annotation>
        <math xmlns="http://www.w3.org/1998/Math/MathML">
          <lambda>
            <bvar>
              <ci> Km </ci>
            </bvar>
            <bvar>
              <ci> Orn </ci>
            </bvar>
            <bvar>
              <ci> V </ci>
            </bvar>
            <apply>
              <divide/>
              <apply>
                <times/>
                <ci> V </ci>
                <ci> Orn </ci>
              </apply>
              <apply>
                <plus/>
                <ci> Km </ci>
                <ci> Orn </ci>
              </apply>
            </apply>
          </lambda>
        </math>
            </functionDefinition>
      <functionDefinition id="Hill_Cooperativity_1_0"
metaid="COPASI35" name="Hill Cooperativity_1">
        <annotation>
          <COPASI xmlns="http://www.copasi.org/static/sbml">
            <rdf:RDF xmlns:dcterms="http://purl.org/dc/terms/"
xmlns:rdf="http://www.w3.org/1999/02/22-rdf-syntax-ns#">
              <rdf:Description rdf:about="#COPASI35">
                <dcterms:created>
                  <rdf:Description>

<dcterms:W3CDTF>2018-03-07T16:42:57Z</dcterms:W3CDTF>
                  </rdf:Description>
```

```
              </dcterms:created>
            </rdf:Description>
          </rdf:RDF>
        </COPASI>
      </annotation>
      <math xmlns="http://www.w3.org/1998/Math/MathML">
        <lambda>
          <bvar>
            <ci> Met </ci>
          </bvar>
          <bvar>
            <ci> Shalve </ci>
          </bvar>
          <bvar>
            <ci> V </ci>
          </bvar>
          <bvar>
            <ci> h </ci>
          </bvar>
          <apply>
            <divide/>
            <apply>
              <times/>
              <ci> V </ci>
              <apply>
                <power/>
                <ci> Met </ci>
                <ci> h </ci>
              </apply>
            </apply>
            <apply>
              <plus/>
              <apply>
                <power/>
                <ci> Shalve </ci>
                <ci> h </ci>
              </apply>
              <apply>
                <power/>
                <ci> Met </ci>
                <ci> h </ci>
              </apply>
            </apply>
          </apply>
        </lambda>
      </math>
          </functionDefinition>
    <functionDefinition
id="Henri_Michaelis_Menten__irreversible__2" metaid="COPASI36"
name="Henri-Michaelis-Menten (irreversible)_2">
      <annotation>
        <COPASI xmlns="http://www.copasi.org/static/sbml">
```

```
            <rdf:RDF xmlns:dcterms="http://purl.org/dc/terms/"
xmlns:rdf="http://www.w3.org/1999/02/22-rdf-syntax-ns#">
              <rdf:Description rdf:about="#COPASI36">
                <dcterms:created>
                  <rdf:Description>

<dcterms:W3CDTF>2018-03-08T12:34:17Z</dcterms:W3CDTF>
                  </rdf:Description>
                </dcterms:created>
              </rdf:Description>
            </rdf:RDF>
          </COPASI>
        </annotation>
        <math xmlns="http://www.w3.org/1998/Math/MathML">
          <lambda>
            <bvar>
              <ci> Arg </ci>
            </bvar>
            <bvar>
              <ci> Km </ci>
            </bvar>
            <bvar>
              <ci> V </ci>
            </bvar>
            <apply>
              <divide/>
              <apply>
                <times/>
                <ci> V </ci>
                <ci> Arg </ci>
              </apply>
              <apply>
                <plus/>
                <ci> Km </ci>
                <ci> Arg </ci>
              </apply>
            </apply>
          </lambda>
        </math>
            </functionDefinition>
      <functionDefinition
id="Henri_Michaelis_Menten__irreversible__1_0" metaid="COPASI37"
name="Henri-Michaelis-Menten (irreversible)_1">
        <annotation>
          <COPASI xmlns="http://www.copasi.org/static/sbml">
            <rdf:RDF xmlns:dcterms="http://purl.org/dc/terms/"
xmlns:rdf="http://www.w3.org/1999/02/22-rdf-syntax-ns#">
              <rdf:Description rdf:about="#COPASI37">
                <dcterms:created>
                  <rdf:Description>

<dcterms:W3CDTF>2018-03-08T12:34:18Z</dcterms:W3CDTF>
```

```
                </rdf:Description>
              </dcterms:created>
            </rdf:Description>
          </rdf:RDF>
        </COPASI>
      </annotation>
      <math xmlns="http://www.w3.org/1998/Math/MathML">
        <lambda>
          <bvar>
            <ci> Km </ci>
          </bvar>
          <bvar>
            <ci> SAM </ci>
          </bvar>
          <bvar>
            <ci> V </ci>
          </bvar>
          <apply>
            <divide/>
            <apply>
              <times/>
              <ci> V </ci>
              <ci> SAM </ci>
            </apply>
            <apply>
              <plus/>
              <ci> Km </ci>
              <ci> SAM </ci>
            </apply>
          </apply>
        </lambda>
      </math>
          </functionDefinition>
    <functionDefinition id="GluCys_1_1" metaid="COPASI38"
name="GluCys_1_1">
      <annotation>
        <COPASI xmlns="http://www.copasi.org/static/sbml">
          <rdf:RDF xmlns:dcterms="http://purl.org/dc/terms/"
xmlns:rdf="http://www.w3.org/1999/02/22-rdf-syntax-ns#">
            <rdf:Description rdf:about="#COPASI38">
              <dcterms:created>
                <rdf:Description>

<dcterms:W3CDTF>2018-01-04T14:24:42Z</dcterms:W3CDTF>
                </rdf:Description>
              </dcterms:created>
            </rdf:Description>
          </rdf:RDF>
        </COPASI>
      </annotation>
      <math xmlns="http://www.w3.org/1998/Math/MathML">
        <lambda>
```

```
    <bvar>
      <ci> GluCys </ci>
    </bvar>
    <bvar>
      <ci> Gly </ci>
    </bvar>
    <bvar>
      <ci> Km1 </ci>
    </bvar>
    <bvar>
      <ci> Km2 </ci>
    </bvar>
    <bvar>
      <ci> Vm </ci>
    </bvar>
    <bvar>
      <ci> kd1 </ci>
    </bvar>
    <apply>
      <divide/>
      <apply>
        <times/>
        <ci> Vm </ci>
        <ci> Gly </ci>
        <ci> GluCys </ci>
      </apply>
      <apply>
        <plus/>
        <apply>
          <times/>
          <ci> Km2 </ci>
          <ci> Gly </ci>
        </apply>
        <apply>
          <times/>
          <ci> Km1 </ci>
          <ci> GluCys </ci>
        </apply>
        <apply>
          <times/>
          <ci> Gly </ci>
          <ci> GluCys </ci>
        </apply>
        <apply>
          <times/>
          <ci> kd1 </ci>
          <ci> Km2 </ci>
        </apply>
      </apply>
    </apply>
  </lambda>
</math>
```


<

```
            </functionDefinition>
      <functionDefinition id="GluCys_1_2" metaid="COPASI39"
name="GluCys_1_2">
        <annotation>
          <COPASI xmlns="http://www.copasi.org/static/sbml">
            <rdf:RDF xmlns:dcterms="http://purl.org/dc/terms/"
xmlns:rdf="http://www.w3.org/1999/02/22-rdf-syntax-ns#">
              <rdf:Description rdf:about="#COPASI39">
                <dcterms:created>
                  <rdf:Description>

<dcterms:W3CDTF>2018-02-27T17:24:04Z</dcterms:W3CDTF>
                  </rdf:Description>
                </dcterms:created>
              </rdf:Description>
            </rdf:RDF>
          </COPASI>
        </annotation>
        <math xmlns="http://www.w3.org/1998/Math/MathML">
          <lambda>
            <bvar>
              <ci> Cys </ci>
            </bvar>
            <bvar>
              <ci> Glu </ci>
            </bvar>
            <bvar>
              <ci> Km1 </ci>
            </bvar>
            <bvar>
              <ci> Km2 </ci>
            </bvar>
            <bvar>
              <ci> Vm </ci>
            </bvar>
            <bvar>
              <ci> kd1 </ci>
            </bvar>
            <apply>
              <divide/>
              <apply>
                <times/>
                <ci> Vm </ci>
                <ci> Cys </ci>
                <ci> Glu </ci>
              </apply>
              <apply>
                <plus/>
                <apply>
                  <times/>
                  <ci> Km2 </ci>
                  <ci> Cys </ci>
```

```
                </apply>
                <apply>
                  <times/>
                  <ci> Km1 </ci>
                  <ci> Glu </ci>
                </apply>
                <apply>
                  <times/>
                  <ci> Cys </ci>
                  <ci> Glu </ci>
                </apply>
                <apply>
                  <times/>
                  <ci> kd1 </ci>
                  <ci> Km2 </ci>
                </apply>
              </apply>
            </apply>
          </lambda>
        </math>
            </functionDefinition>
      <functionDefinition id="Bi__irreversible__1"
metaid="COPASI40" name="Bi (irreversible)_1">
        <annotation>
          <COPASI xmlns="http://www.copasi.org/static/sbml">
            <rdf:RDF xmlns:dcterms="http://purl.org/dc/terms/"
xmlns:rdf="http://www.w3.org/1999/02/22-rdf-syntax-ns#">
              <rdf:Description rdf:about="#COPASI40">
                <dcterms:created>
                  <rdf:Description>

<dcterms:W3CDTF>2018-01-04T10:58:50Z</dcterms:W3CDTF>
                  </rdf:Description>
                </dcterms:created>
              </rdf:Description>
            </rdf:RDF>
          </COPASI>
        </annotation>
        <math xmlns="http://www.w3.org/1998/Math/MathML">
          <lambda>
            <bvar>
              <ci> GSH </ci>
            </bvar>
            <bvar>
              <ci> Kma </ci>
            </bvar>
            <bvar>
              <ci> Kmb </ci>
            </bvar>
            <bvar>
              <ci> Spd </ci>
            </bvar>
```

```
            <bvar>
              <ci> vmax </ci>
            </bvar>
            <apply>
              <divide/>
              <apply>
                <times/>
                <ci> vmax </ci>
                <ci> Spd </ci>
                <ci> GSH </ci>
              </apply>
              <apply>
                <plus/>
                <apply>
                  <times/>
                  <ci> Kma </ci>
                  <ci> Kmb </ci>
                </apply>
                <apply>
                  <times/>
                  <ci> Spd </ci>
                  <ci> Kmb </ci>
                </apply>
                <apply>
                  <times/>
                  <ci> GSH </ci>
                  <ci> Kma </ci>
                </apply>
                <apply>
                  <times/>
                  <ci> Spd </ci>
                  <ci> GSH </ci>
                </apply>
              </apply>
            </apply>
          </lambda>
        </math>
            </functionDefinition>
      <functionDefinition id="BiBiPingPong_Enzyme"
metaid="COPASI41" name="BiBiPingPong_Enzyme">
        <annotation>
          <COPASI xmlns="http://www.copasi.org/static/sbml">
            <rdf:RDF xmlns:dcterms="http://purl.org/dc/terms/"
xmlns:rdf="http://www.w3.org/1999/02/22-rdf-syntax-ns#">
              <rdf:Description rdf:about="#COPASI41">
                <dcterms:created>
                  <rdf:Description>

<dcterms:W3CDTF>2018-03-12T15:08:54Z</dcterms:W3CDTF>
                  </rdf:Description>
                </dcterms:created>
              </rdf:Description>
```

```
    </rdf:RDF>
  </COPASI>
</annotation>
<math xmlns="http://www.w3.org/1998/Math/MathML">
  <lambda>
    <bvar>
      <ci> Kcat </ci>
    </bvar>
    <bvar>
      <ci> E </ci>
    </bvar>
    <bvar>
      <ci> A </ci>
    </bvar>
    <bvar>
      <ci> B </ci>
    </bvar>
    <bvar>
      <ci> Kb </ci>
    </bvar>
    <bvar>
      <ci> Ka </ci>
    </bvar>
    <apply>
      <divide/>
      <apply>
        <times/>
        <ci> Kcat </ci>
        <ci> E </ci>
        <ci> A </ci>
        <ci> B </ci>
      </apply>
      <apply>
        <plus/>
        <apply>
          <times/>
          <ci> Kb </ci>
          <ci> A </ci>
        </apply>
        <apply>
          <times/>
          <ci> Ka </ci>
          <ci> B </ci>
        </apply>
        <apply>
          <times/>
          <ci> A </ci>
          <ci> B </ci>
        </apply>
      </apply>
    </apply>
  </lambda>
```

```
        </math>
            </functionDefinition>
      <functionDefinition id="Two_sub" metaid="COPASI42"
name="Two sub">
        <annotation>
          <COPASI xmlns="http://www.copasi.org/static/sbml">
            <rdf:RDF xmlns:dcterms="http://purl.org/dc/terms/"
xmlns:rdf="http://www.w3.org/1999/02/22-rdf-syntax-ns#">
              <rdf:Description rdf:about="#COPASI42">
                <dcterms:created>
                  <rdf:Description>

<dcterms:W3CDTF>2018-07-10T16:43:25Z</dcterms:W3CDTF>
                  </rdf:Description>
                </dcterms:created>
              </rdf:Description>
            </rdf:RDF>
          </COPASI>
        </annotation>
        <math xmlns="http://www.w3.org/1998/Math/MathML">
          <lambda>
            <bvar>
              <ci> V </ci>
            </bvar>
            <bvar>
              <ci> A </ci>
            </bvar>
            <bvar>
              <ci> B </ci>
            </bvar>
            <bvar>
              <ci> KmA </ci>
            </bvar>
            <bvar>
              <ci> KmB </ci>
            </bvar>
            <apply>
              <divide/>
              <apply>
                <times/>
                <ci> V </ci>
                <ci> A </ci>
                <ci> B </ci>
              </apply>
              <apply>
                <plus/>
                <apply>
                  <times/>
                  <ci> KmA </ci>
                  <ci> KmB </ci>
                </apply>
                <apply>
```

```
                <times/>
                <ci> KmB </ci>
                <ci> A </ci>
              </apply>
              <apply>
                <times/>
                <ci> KmA </ci>
                <ci> B </ci>
              </apply>
            </apply>
          </apply>
        </lambda>
      </math>
          </functionDefinition>
  </listOfFunctionDefinitions>
  <listOfCompartments>
    <compartment constant="true" id="cell" name="cell" size="1"
spatialDimensions="3"/>
  </listOfCompartments>
  <listOfSpecies>
    <species boundaryCondition="false" compartment="cell"
constant="false" id="Met"
initialConcentration="9.99999999999983E-6" name="Met"/>
    <species boundaryCondition="false" compartment="cell"
constant="false" id="SAM" initialConcentration="0" name="SAM"/>
    <species boundaryCondition="false" compartment="cell"
constant="false" id="dcSAM" initialConcentration="0"
name="dcSAM"/>
    <species boundaryCondition="false" compartment="cell"
constant="false" id="Arg"
initialConcentration="0.00199999999999995" name="Arg"/>
    <species boundaryCondition="false" compartment="cell"
constant="false" id="Orn" initialConcentration="0" name="Orn"/>
    <species boundaryCondition="false" compartment="cell"
constant="false" id="Put" initialConcentration="0" name="Put"/>
    <species boundaryCondition="false" compartment="cell"
constant="false" id="Spd" initialConcentration="0" name="Spd"/>
    <species boundaryCondition="false" compartment="cell"
constant="false" id="Cys"
initialConcentration="9.99999999999983E-6" name="Cys"/>
    <species boundaryCondition="false" compartment="cell"
constant="false" id="Glu"
initialConcentration="9.99999999999983E-6" name="Glu"/>
    <species boundaryCondition="false" compartment="cell"
constant="false" id="GluCys" initialConcentration="0"
name="GluCys"/>
    <species boundaryCondition="false" compartment="cell"
constant="false" id="GSH" initialConcentration="0" name="GSH"/>
    <species boundaryCondition="false" compartment="cell"
constant="false" id="Gly"
initialConcentration="9.99999999999983E-6" name="Gly"/>
    <species boundaryCondition="false" compartment="cell"
```

```
constant="false" id="Gspd" initialConcentration="0" name="Gspd"/>
      <species boundaryCondition="false" compartment="cell"
constant="false" id="TS2"
initialConcentration="4.99999999999973E-5" name="TS2"/>
      <species boundaryCondition="false" compartment="cell"
constant="false" id="T_SH_2" initialConcentration="0"
name="T[SH]2"/>
      <species boundaryCondition="false" compartment="cell"
constant="false" id="CO2" initialConcentration="0" name="CO2"/>
      <species boundaryCondition="false" compartment="cell"
constant="false" id="TXNo" initialConcentration="0" name="TXNo"/>
      <species boundaryCondition="false" compartment="cell"
constant="false" id="TXNr"
initialConcentration="2.79999999999985E-5" name="TXNr"/>
      <species boundaryCondition="false" compartment="cell"
constant="false" id="H2O" initialConcentration="0"
metaid="COPASI1" name="H2O">
        <annotation>
          <COPASI xmlns="http://www.copasi.org/static/sbml">
            <rdf:RDF xmlns:dcterms="http://purl.org/dc/terms/"
xmlns:rdf="http://www.w3.org/1999/02/22-rdf-syntax-ns#">
              <rdf:Description rdf:about="#COPASI1">
                <dcterms:created>
                  <rdf:Description>

<dcterms:W3CDTF>2018-07-09T11:40:27Z</dcterms:W3CDTF>
                  </rdf:Description>
                </dcterms:created>
              </rdf:Description>
            </rdf:RDF>
          </COPASI>
        </annotation>
            </species>
      <species boundaryCondition="false" compartment="cell"
constant="false" id="H2O2"
initialConcentration="0.000299999999999999" name="H2O2"/>
      <species boundaryCondition="false" compartment="cell"
constant="false" id="TDPx" initialConcentration="2E-7"
name="TDPx"/>
      <species boundaryCondition="false" compartment="cell"
constant="false" id="TryP"
initialConcentration="1.99999999999989E-7" name="TryP"/>
      <species boundaryCondition="true" compartment="cell"
constant="true" id="Fe_2"
initialConcentration="8.79999999999967E-9" name="Fe+2"/>
      <species boundaryCondition="false" compartment="cell"
constant="false" id="aconitase"
initialConcentration="6.69999999999982E-6" name="aconitase"/>
      <species boundaryCondition="false" compartment="cell"
constant="false" id="fumarase_4Fe_4S" initialConcentration="0"
name="fumarase[4Fe-4S]"/>
      <species boundaryCondition="false" compartment="cell"
```

```
constant="false" id="Fumarase"
initialConcentration="6.69999999999986E-6" name="Fumarase"/>
      <species boundaryCondition="false" compartment="cell"
constant="false" id="Grx1"
initialConcentration="1.18999999999999E-5" name="Grx1"/>
      <species boundaryCondition="false" compartment="cell"
constant="false" id="Grx1r" initialConcentration="0"
metaid="COPASI2" name="Grx1r">
        <annotation>
          <COPASI xmlns="http://www.copasi.org/static/sbml">
            <rdf:RDF xmlns:dcterms="http://purl.org/dc/terms/"
xmlns:rdf="http://www.w3.org/1999/02/22-rdf-syntax-ns#">
              <rdf:Description rdf:about="#COPASI2">
                <dcterms:created>
                  <rdf:Description>

<dcterms:W3CDTF>2018-07-10T15:15:12Z</dcterms:W3CDTF>
                  </rdf:Description>
                </dcterms:created>
              </rdf:Description>
            </rdf:RDF>
          </COPASI>
        </annotation>
            </species>
      <species boundaryCondition="false" compartment="cell"
constant="false" id="Grx2"
initialConcentration="1.37999999999997E-5" name="Grx2"/>
      <species boundaryCondition="false" compartment="cell"
constant="false" id="Grx2r" initialConcentration="0"
name="Grx2r"/>
      <species boundaryCondition="false" compartment="cell"
constant="false" id="aconitase_4Fe_4S" initialConcentration="0"
name="aconitase[4Fe-4S]"/>
      <species boundaryCondition="false" compartment="cell"
constant="false" id="ISCU"
initialConcentration="1.99999999999997E-5" name="ISCU"/>
      <species boundaryCondition="false" compartment="cell"
constant="false" id="ISCU_2Fe_2S" initialConcentration="0"
name="ISCU[2Fe-2S]"/>
      <species boundaryCondition="false" compartment="cell"
constant="false" id="ISCU_2S"
initialConcentration="1.99999999999997E-5" name="ISCU[2S]"/>
      <species boundaryCondition="false" compartment="cell"
constant="false" id="ISC_Grx1r_4Fe_4S" initialConcentration="0"
name="ISC-Grx1r[4Fe-4S]"/>
      <species boundaryCondition="false" compartment="cell"
constant="false" id="ISC_Grx2r_4Fe_4S" initialConcentration="0"
name="ISC-Grx2r[4Fe-4S]"/>
      <species boundaryCondition="false" compartment="cell"
constant="false" id="O2_"
initialConcentration="9.9999999999999E-9" name="O2.-"/>
      <species boundaryCondition="false" compartment="cell"
```

```
constant="false" id="_OH" initialConcentration="0" name=".OH"/>
      <species boundaryCondition="false" compartment="cell"
constant="false" id="Fe_3" initialConcentration="0" name="Fe+3"/>
      <species boundaryCondition="false" compartment="cell"
constant="false" id="OH" initialConcentration="0" name="OH-"/>
      <species boundaryCondition="false" compartment="cell"
constant="false" id="aconitase_3Fe_4S_" initialConcentration="0"
name="aconitase[3Fe-4S]+"/>
      <species boundaryCondition="false" compartment="cell"
constant="false" id="fumarase_3Fe_4S_" initialConcentration="0"
name="fumarase[3Fe-4S]+"/>
      <species boundaryCondition="false" compartment="cell"
constant="false" id="ONOO"
initialConcentration="0.000199999999999999" name="ONOO-"/>
      <species boundaryCondition="false" compartment="cell"
constant="false" id="NADP" initialConcentration="0" name="NADP"/>
      <species boundaryCondition="false" compartment="cell"
constant="false" id="NADPH"
initialConcentration="7.99999999999999E-5" name="NADPH"/>
    </listOfSpecies>
    <listOfReactions>
      <reaction id="SAMsyn" metaid="COPASI3" name="SAMsyn"
reversible="false">
        <annotation>
          <COPASI xmlns="http://www.copasi.org/static/sbml">
            <rdf:RDF xmlns:dcterms="http://purl.org/dc/terms/"
xmlns:rdf="http://www.w3.org/1999/02/22-rdf-syntax-ns#">
              <rdf:Description rdf:about="#COPASI3">
                <dcterms:created>
                  <rdf:Description>

<dcterms:W3CDTF>2017-06-28T16:21:21Z</dcterms:W3CDTF>
                  </rdf:Description>
                </dcterms:created>
              </rdf:Description>
            </rdf:RDF>
          </COPASI>
        </annotation>
              <listOfReactants>
          <speciesReference species="Met" stoichiometry="1"/>
        </listOfReactants>
        <listOfProducts>
          <speciesReference species="SAM" stoichiometry="1"/>
        </listOfProducts>
        <kineticLaw>
          <math xmlns="http://www.w3.org/1998/Math/MathML">
            <apply>
              <times/>
              <ci> cell </ci>
              <apply>
                <ci> Hill_Cooperativity_1_0 </ci>
                <ci> Met </ci>
```

```
                <ci> Shalve </ci>
                <ci> V </ci>
                <ci> h </ci>
              </apply>
            </apply>
          </math>
                <listOfParameters>
            <parameter id="Shalve" name="Shalve"
value="0.00025"/>
            <parameter id="V" name="V" value="3.4E-9"/>
            <parameter id="h" name="h" value="2.3"/>
          </listOfParameters>
        </kineticLaw>
      </reaction>
      <reaction id="SAMdc" metaid="COPASI4" name="SAMdc"
reversible="false">
        <annotation>
          <COPASI xmlns="http://www.copasi.org/static/sbml">
            <rdf:RDF xmlns:dcterms="http://purl.org/dc/terms/"
xmlns:rdf="http://www.w3.org/1999/02/22-rdf-syntax-ns#">
              <rdf:Description rdf:about="#COPASI4">
                <dcterms:created>
                  <rdf:Description>

<dcterms:W3CDTF>2017-06-28T16:27:31Z</dcterms:W3CDTF>
                  </rdf:Description>
                </dcterms:created>
              </rdf:Description>
            </rdf:RDF>
          </COPASI>
        </annotation>
              <listOfReactants>
          <speciesReference species="SAM" stoichiometry="1"/>
        </listOfReactants>
        <listOfProducts>
          <speciesReference species="dcSAM" stoichiometry="1"/>
        </listOfProducts>
        <kineticLaw>
          <math xmlns="http://www.w3.org/1998/Math/MathML">
            <apply>
              <times/>
              <ci> cell </ci>
              <apply>
                <ci> Henri_Michaelis_Menten__irreversible__1_0
</ci>
                <ci> Km </ci>
                <ci> SAM </ci>
                <ci> V </ci>
              </apply>
            </apply>
          </math>
                <listOfParameters>
```

```
            <parameter id="Km" name="Km" value="0.00038"/>
            <parameter id="V" name="V" value="5.2E-9"/>
          </listOfParameters>
        </kineticLaw>
      </reaction>
      <reaction id="Arginase" metaid="COPASI5" name="Arginase"
reversible="false">
        <annotation>
          <COPASI xmlns="http://www.copasi.org/static/sbml">
            <rdf:RDF xmlns:dcterms="http://purl.org/dc/terms/"
xmlns:rdf="http://www.w3.org/1999/02/22-rdf-syntax-ns#">
              <rdf:Description rdf:about="#COPASI5">
                <dcterms:created>
                  <rdf:Description>

<dcterms:W3CDTF>2017-06-28T17:25:21Z</dcterms:W3CDTF>
                  </rdf:Description>
                </dcterms:created>
              </rdf:Description>
            </rdf:RDF>
          </COPASI>
        </annotation>
              <listOfReactants>
          <speciesReference species="Arg" stoichiometry="1"/>
        </listOfReactants>
        <listOfProducts>
          <speciesReference species="Orn" stoichiometry="1"/>
        </listOfProducts>
        <kineticLaw>
          <math xmlns="http://www.w3.org/1998/Math/MathML">
            <apply>
              <times/>
              <ci> cell </ci>
              <apply>
                <ci> Henri_Michaelis_Menten__irreversible__2
</ci>
                <ci> Arg </ci>
                <ci> Km </ci>
                <ci> V </ci>
              </apply>
            </apply>
          </math>
                  <listOfParameters>
            <parameter id="Km" name="Km" value="0.0215"/>
            <parameter id="V" name="V" value="0.0024"/>
          </listOfParameters>
        </kineticLaw>
      </reaction>
      <reaction id="ODC" metaid="COPASI6" name="ODC"
reversible="false">
        <annotation>
          <COPASI xmlns="http://www.copasi.org/static/sbml">
```

```
            <rdf:RDF xmlns:dcterms="http://purl.org/dc/terms/"
xmlns:rdf="http://www.w3.org/1999/02/22-rdf-syntax-ns#">
              <rdf:Description rdf:about="#COPASI6">
                <dcterms:created>
                  <rdf:Description>

<dcterms:W3CDTF>2017-06-28T17:26:39Z</dcterms:W3CDTF>
                  </rdf:Description>
                </dcterms:created>
              </rdf:Description>
            </rdf:RDF>
          </COPASI>
        </annotation>
              <listOfReactants>
          <speciesReference species="Orn" stoichiometry="1"/>
        </listOfReactants>
        <listOfProducts>
          <speciesReference species="Put" stoichiometry="1"/>
          <speciesReference species="CO2" stoichiometry="1"/>
        </listOfProducts>
        <kineticLaw>
          <math xmlns="http://www.w3.org/1998/Math/MathML">
            <apply>
              <times/>
              <ci> cell </ci>
              <apply>
                <ci> Henri_Michaelis_Menten__irreversible__3
</ci>
                <ci> Km </ci>
                <ci> Orn </ci>
                <ci> V </ci>
              </apply>
            </apply>
          </math>
                  <listOfParameters>
            <parameter id="Km" name="Km" value="0.00042"/>
            <parameter id="V" name="V" value="0.025256"/>
          </listOfParameters>
        </kineticLaw>
      </reaction>
      <reaction id="SpdS" metaid="COPASI7" name="SpdS"
reversible="false">
        <annotation>
          <COPASI xmlns="http://www.copasi.org/static/sbml">
            <rdf:RDF xmlns:dcterms="http://purl.org/dc/terms/"
xmlns:rdf="http://www.w3.org/1999/02/22-rdf-syntax-ns#">
              <rdf:Description rdf:about="#COPASI7">
                <dcterms:created>
                  <rdf:Description>

<dcterms:W3CDTF>2017-06-28T17:28:48Z</dcterms:W3CDTF>
                  </rdf:Description>
```

```
              </dcterms:created>
            </rdf:Description>
          </rdf:RDF>
        </COPASI>
      </annotation>
            <listOfReactants>
        <speciesReference species="Put" stoichiometry="1"/>
        <speciesReference species="dcSAM" stoichiometry="1"/>
      </listOfReactants>
      <listOfProducts>
        <speciesReference species="Spd" stoichiometry="1"/>
      </listOfProducts>
      <kineticLaw>
        <math xmlns="http://www.w3.org/1998/Math/MathML">
          <apply>
            <times/>
            <ci> cell </ci>
            <apply>
              <ci> SpdSynth_1 </ci>
              <ci> KddcSAM </ci>
              <ci> KmPut </ci>
              <ci> KmdcSAM </ci>
              <ci> Put </ci>
              <ci> VmPut </ci>
              <ci> dcSAM </ci>
            </apply>
          </apply>
        </math>
                <listOfParameters>
          <parameter id="KddcSAM" name="KddcSAM" value="0.1"/>
          <parameter id="KmPut" name="KmPut" value="0.000205"/>
          <parameter id="KmdcSAM" name="KmdcSAM" value="9E-8"/>
          <parameter id="VmPut" name="VmPut" value="1.98E-13"/>
        </listOfParameters>
      </kineticLaw>
    </reaction>
    <reaction id="gECS" metaid="COPASI8" name="yECS"
reversible="false">
      <annotation>
        <COPASI xmlns="http://www.copasi.org/static/sbml">
          <rdf:RDF xmlns:dcterms="http://purl.org/dc/terms/"
xmlns:rdf="http://www.w3.org/1999/02/22-rdf-syntax-ns#">
            <rdf:Description rdf:about="#COPASI8">
              <dcterms:created>
                <rdf:Description>

<dcterms:W3CDTF>2017-07-04T15:52:19Z</dcterms:W3CDTF>
                </rdf:Description>
              </dcterms:created>
            </rdf:Description>
          </rdf:RDF>
        </COPASI>
```

```
        </annotation>
              <listOfReactants>
          <speciesReference species="Glu" stoichiometry="1"/>
          <speciesReference species="Cys" stoichiometry="1"/>
        </listOfReactants>
        <listOfProducts>
          <speciesReference species="GluCys" stoichiometry="1"/>
        </listOfProducts>
        <kineticLaw>
          <math xmlns="http://www.w3.org/1998/Math/MathML">
            <apply>
              <times/>
              <ci> cell </ci>
              <apply>
                <ci> GluCys_1_2 </ci>
                <ci> Cys </ci>
                <ci> Glu </ci>
                <ci> Km1 </ci>
                <ci> Km2 </ci>
                <ci> Vm </ci>
                <ci> kd1 </ci>
              </apply>
            </apply>
          </math>
                <listOfParameters>
            <parameter id="Km1" name="Km1" value="0.0092"/>
            <parameter id="Km2" name="Km2" value="0.0017"/>
            <parameter id="Vm" name="Vm" value="1.8E-6"/>
            <parameter id="kd1" name="kd1" value="9.37E-5"/>
          </listOfParameters>
        </kineticLaw>
      </reaction>
      <reaction id="GS" metaid="COPASI9" name="GS"
reversible="false">
        <annotation>
          <COPASI xmlns="http://www.copasi.org/static/sbml">
            <rdf:RDF xmlns:dcterms="http://purl.org/dc/terms/"
xmlns:rdf="http://www.w3.org/1999/02/22-rdf-syntax-ns#">
              <rdf:Description rdf:about="#COPASI9">
                <dcterms:created>
                  <rdf:Description>

<dcterms:W3CDTF>2017-07-04T15:56:04Z</dcterms:W3CDTF>
                  </rdf:Description>
                </dcterms:created>
              </rdf:Description>
            </rdf:RDF>
          </COPASI>
        </annotation>
              <listOfReactants>
          <speciesReference species="GluCys" stoichiometry="1"/>
          <speciesReference species="Gly" stoichiometry="1"/>
```

```
        </listOfReactants>
        <listOfProducts>
          <speciesReference species="GSH" stoichiometry="1"/>
        </listOfProducts>
        <kineticLaw>
          <math xmlns="http://www.w3.org/1998/Math/MathML">
            <apply>
              <times/>
              <ci> cell </ci>
              <apply>
                <ci> GluCys_1_1 </ci>
                <ci> GluCys </ci>
                <ci> Gly </ci>
                <ci> Km1 </ci>
                <ci> Km2 </ci>
                <ci> Vm </ci>
                <ci> kd1 </ci>
              </apply>
            </apply>
          </math>
                  <listOfParameters>
            <parameter id="Km1" name="Km1" value="4E-5"/>
            <parameter id="Km2" name="Km2" value="0.0012"/>
            <parameter id="Vm" name="Vm" value="3.4E-8"/>
            <parameter id="kd1" name="kd1" value="8E-5"/>
          </listOfParameters>
        </kineticLaw>
      </reaction>
      <reaction id="TryS1" metaid="COPASI10" name="TryS1"
reversible="false">
        <annotation>
          <COPASI xmlns="http://www.copasi.org/static/sbml">
            <rdf:RDF xmlns:dcterms="http://purl.org/dc/terms/"
xmlns:rdf="http://www.w3.org/1999/02/22-rdf-syntax-ns#">
              <rdf:Description rdf:about="#COPASI10">
                <dcterms:created>
                  <rdf:Description>

<dcterms:W3CDTF>2017-07-04T16:01:02Z</dcterms:W3CDTF>
                  </rdf:Description>
                </dcterms:created>
              </rdf:Description>
            </rdf:RDF>
          </COPASI>
        </annotation>
              <listOfReactants>
          <speciesReference species="Spd" stoichiometry="1"/>
          <speciesReference species="GSH" stoichiometry="1"/>
        </listOfReactants>
        <listOfProducts>
          <speciesReference species="Gspd" stoichiometry="1"/>
        </listOfProducts>
```

```
        <kineticLaw>
          <math xmlns="http://www.w3.org/1998/Math/MathML">
            <apply>
              <times/>
              <ci> cell </ci>
              <apply>
                <ci> Bi__irreversible__1 </ci>
                <ci> GSH </ci>
                <ci> Kma </ci>
                <ci> Kmb </ci>
                <ci> Spd </ci>
                <ci> vmax </ci>
              </apply>
            </apply>
          </math>
                  <listOfParameters>
            <parameter id="Kma" name="Kma" value="0.00094"/>
            <parameter id="Kmb" name="Kmb" value="8.9E-5"/>
            <parameter id="vmax" name="vmax" value="8E-7"/>
          </listOfParameters>
        </kineticLaw>
      </reaction>
      <reaction id="TryS2" metaid="COPASI11" name="TryS2"
reversible="false">
        <annotation>
          <COPASI xmlns="http://www.copasi.org/static/sbml">
            <rdf:RDF xmlns:dcterms="http://purl.org/dc/terms/"
xmlns:rdf="http://www.w3.org/1999/02/22-rdf-syntax-ns#">
              <rdf:Description rdf:about="#COPASI11">
                <dcterms:created>
                  <rdf:Description>

<dcterms:W3CDTF>2017-07-05T15:34:28Z</dcterms:W3CDTF>
                  </rdf:Description>
                </dcterms:created>
              </rdf:Description>
            </rdf:RDF>
          </COPASI>
        </annotation>
              <listOfReactants>
          <speciesReference species="Gspd" stoichiometry="1"/>
          <speciesReference species="GSH" stoichiometry="1"/>
        </listOfReactants>
        <listOfProducts>
          <speciesReference species="T_SH_2" stoichiometry="1"/>
        </listOfProducts>
        <kineticLaw>
          <math xmlns="http://www.w3.org/1998/Math/MathML">
            <apply>
              <times/>
              <ci> cell </ci>
              <apply>
```

```
                <ci> Bi__irreversible </ci>
                <ci> vmax </ci>
                <ci> Gspd </ci>
                <ci> GSH </ci>
                <ci> Kma </ci>
                <ci> Kmb </ci>
              </apply>
            </apply>
          </math>
                <listOfParameters>
            <parameter id="vmax" name="vmax" value="8E-7"/>
            <parameter id="Kma" name="Kma" value="4E-5"/>
            <parameter id="Kmb" name="Kmb" value="8.9E-5"/>
          </listOfParameters>
        </kineticLaw>
      </reaction>
      <reaction id="TR" metaid="COPASI12" name="TR"
reversible="false">
        <annotation>
          <COPASI xmlns="http://www.copasi.org/static/sbml">
            <rdf:RDF xmlns:dcterms="http://purl.org/dc/terms/"
xmlns:rdf="http://www.w3.org/1999/02/22-rdf-syntax-ns#">
              <rdf:Description rdf:about="#COPASI12">
                <dcterms:created>
                  <rdf:Description>

<dcterms:W3CDTF>2018-03-07T16:29:32Z</dcterms:W3CDTF>
                  </rdf:Description>
                </dcterms:created>
              </rdf:Description>
            </rdf:RDF>
          </COPASI>
        </annotation>
              <listOfReactants>
          <speciesReference species="TS2" stoichiometry="1"/>
          <speciesReference species="NADPH" stoichiometry="1"/>
        </listOfReactants>
        <listOfProducts>
          <speciesReference species="T_SH_2" stoichiometry="1"/>
          <speciesReference species="NADP" stoichiometry="1"/>
        </listOfProducts>
        <kineticLaw>
          <math xmlns="http://www.w3.org/1998/Math/MathML">
            <apply>
              <times/>
              <ci> cell </ci>
              <apply>
                <ci> Two_sub </ci>
                <ci> V </ci>
                <ci> TS2 </ci>
                <ci> NADPH </ci>
                <ci> KmA </ci>
```

```
                <ci> KmB </ci>
              </apply>
            </apply>
          </math>
                <listOfParameters>
            <parameter id="V" name="V" value="3.33E-6"/>
            <parameter id="KmA" name="KmA" value="5E-5"/>
            <parameter id="KmB" name="KmB" value="2E-5"/>
          </listOfParameters>
        </kineticLaw>
      </reaction>
      <reaction id="TXNo_Reduction" metaid="COPASI13" name="TXNo
Reduction" reversible="false">
        <annotation>
          <COPASI xmlns="http://www.copasi.org/static/sbml">
            <rdf:RDF xmlns:dcterms="http://purl.org/dc/terms/"
xmlns:rdf="http://www.w3.org/1999/02/22-rdf-syntax-ns#">
              <rdf:Description rdf:about="#COPASI13">
                <dcterms:created>
                  <rdf:Description>

<dcterms:W3CDTF>2018-03-12T15:07:28Z</dcterms:W3CDTF>
                  </rdf:Description>
                </dcterms:created>
              </rdf:Description>
            </rdf:RDF>
          </COPASI>
        </annotation>
              <listOfReactants>
          <speciesReference species="T_SH_2" stoichiometry="1"/>
          <speciesReference species="TXNo" stoichiometry="1"/>
        </listOfReactants>
        <listOfProducts>
          <speciesReference species="TXNr" stoichiometry="1"/>
          <speciesReference species="TS2" stoichiometry="1"/>
        </listOfProducts>
        <kineticLaw>
          <math xmlns="http://www.w3.org/1998/Math/MathML">
            <apply>
              <times/>
              <ci> cell </ci>
              <ci> k1 </ci>
              <ci> T_SH_2 </ci>
              <ci> TXNo </ci>
            </apply>
          </math>
                <listOfParameters>
            <parameter id="k1" name="k1" value="200"/>
          </listOfParameters>
        </kineticLaw>
      </reaction>
      <reaction id="TDPx_0" metaid="COPASI14" name="TDPx"
```

```
reversible="false">
        <annotation>
          <COPASI xmlns="http://www.copasi.org/static/sbml">
            <rdf:RDF xmlns:dcterms="http://purl.org/dc/terms/"
xmlns:rdf="http://www.w3.org/1999/02/22-rdf-syntax-ns#">
              <rdf:Description rdf:about="#COPASI14">
                <dcterms:created>
                  <rdf:Description>

<dcterms:W3CDTF>2018-03-12T15:08:13Z</dcterms:W3CDTF>
                  </rdf:Description>
                </dcterms:created>
              </rdf:Description>
            </rdf:RDF>
          </COPASI>
        </annotation>
              <listOfReactants>
          <speciesReference species="H2O2" stoichiometry="1"/>
          <speciesReference species="TXNr" stoichiometry="1"/>
        </listOfReactants>
        <listOfProducts>
          <speciesReference species="TXNo" stoichiometry="1"/>
          <speciesReference species="H2O" stoichiometry="1"/>
        </listOfProducts>
        <listOfModifiers>
          <modifierSpeciesReference species="TDPx"/>
        </listOfModifiers>
        <kineticLaw>
          <math xmlns="http://www.w3.org/1998/Math/MathML">
            <apply>
              <times/>
              <ci> cell </ci>
              <apply>
                <ci> BiBiPingPong_Enzyme </ci>
                <ci> Kcat </ci>
                <ci> TDPx </ci>
                <ci> H2O2 </ci>
                <ci> TXNr </ci>
                <ci> Kb </ci>
                <ci> Ka </ci>
              </apply>
            </apply>
          </math>
                  <listOfParameters>
            <parameter id="Kcat" name="Kcat" value="15.4"/>
            <parameter id="Kb" name="Kb" value="2.2E-6"/>
            <parameter id="Ka" name="Ka" value="0.000193"/>
          </listOfParameters>
        </kineticLaw>
      </reaction>
      <reaction id="TryP_0" metaid="COPASI15" name="TryP"
reversible="false">
```

```
        <annotation>
          <COPASI xmlns="http://www.copasi.org/static/sbml">
            <rdf:RDF xmlns:dcterms="http://purl.org/dc/terms/"
xmlns:rdf="http://www.w3.org/1999/02/22-rdf-syntax-ns#">
              <rdf:Description rdf:about="#COPASI15">
                <dcterms:created>
                  <rdf:Description>

<dcterms:W3CDTF>2018-03-12T15:13:05Z</dcterms:W3CDTF>
                  </rdf:Description>
                </dcterms:created>
              </rdf:Description>
            </rdf:RDF>
          </COPASI>
        </annotation>
              <listOfReactants>
          <speciesReference species="H2O2" stoichiometry="1"/>
          <speciesReference species="TXNr" stoichiometry="1"/>
        </listOfReactants>
        <listOfProducts>
          <speciesReference species="TXNo" stoichiometry="1"/>
          <speciesReference species="H2O" stoichiometry="1"/>
        </listOfProducts>
        <listOfModifiers>
          <modifierSpeciesReference species="TryP"/>
        </listOfModifiers>
        <kineticLaw>
          <math xmlns="http://www.w3.org/1998/Math/MathML">
            <apply>
              <times/>
              <ci> cell </ci>
              <apply>
                <ci> BiBiPingPong_Enzyme </ci>
                <ci> Kcat </ci>
                <ci> TryP </ci>
                <ci> H2O2 </ci>
                <ci> TXNr </ci>
                <ci> Kb </ci>
                <ci> Ka </ci>
              </apply>
            </apply>
          </math>
                  <listOfParameters>
            <parameter id="Kcat" name="Kcat" value="8.8"/>
            <parameter id="Kb" name="Kb" value="4.9E-6"/>
            <parameter id="Ka" name="Ka" value="6.3E-6"/>
          </listOfParameters>
        </kineticLaw>
      </reaction>
      <reaction id="ISCU_activation" metaid="COPASI16" name="ISCU
activation" reversible="false">
        <annotation>
```

```
          <COPASI xmlns="http://www.copasi.org/static/sbml">
            <rdf:RDF xmlns:dcterms="http://purl.org/dc/terms/"
xmlns:rdf="http://www.w3.org/1999/02/22-rdf-syntax-ns#">
              <rdf:Description rdf:about="#COPASI16">
                <dcterms:created>
                  <rdf:Description>

<dcterms:W3CDTF>2018-07-09T11:37:18Z</dcterms:W3CDTF>
                  </rdf:Description>
                </dcterms:created>
              </rdf:Description>
            </rdf:RDF>
          </COPASI>
        </annotation>
              <listOfReactants>
          <speciesReference species="Fe_2" stoichiometry="2"/>
          <speciesReference species="ISCU_2S" stoichiometry="1"/>
        </listOfReactants>
        <listOfProducts>
          <speciesReference species="ISCU_2Fe_2S"
stoichiometry="1"/>
        </listOfProducts>
        <kineticLaw>
          <math xmlns="http://www.w3.org/1998/Math/MathML">
            <apply>
              <times/>
              <ci> cell </ci>
              <apply>
                <ci> Two_sub </ci>
                <ci> V </ci>
                <ci> Fe_2 </ci>
                <ci> Fe_2 </ci>
                <ci> KmA </ci>
                <ci> KmB </ci>
              </apply>
            </apply>
          </math>
                  <listOfParameters>
            <parameter id="V" name="V" value="0.01109"/>
            <parameter id="KmA" name="KmA" value="3.612E-5"/>
            <parameter id="KmB" name="KmB" value="1E-6"/>
          </listOfParameters>
        </kineticLaw>
      </reaction>
      <reaction id="Aco_activation_grx1" metaid="COPASI17"
name="Aco_activation_grx1" reversible="false">
        <annotation>
          <COPASI xmlns="http://www.copasi.org/static/sbml">
            <rdf:RDF xmlns:dcterms="http://purl.org/dc/terms/"
xmlns:rdf="http://www.w3.org/1999/02/22-rdf-syntax-ns#">
              <rdf:Description rdf:about="#COPASI17">
                <dcterms:created>
```

```
                  <rdf:Description>

<dcterms:W3CDTF>2018-07-09T11:42:34Z</dcterms:W3CDTF>
                  </rdf:Description>
                </dcterms:created>
              </rdf:Description>
            </rdf:RDF>
          </COPASI>
        </annotation>
              <listOfReactants>
          <speciesReference species="aconitase"
stoichiometry="1"/>
          <speciesReference species="ISC_Grx1r_4Fe_4S"
stoichiometry="1"/>
        </listOfReactants>
        <listOfProducts>
          <speciesReference species="aconitase_4Fe_4S"
stoichiometry="1"/>
          <speciesReference species="ISCU" stoichiometry="1"/>
        </listOfProducts>
        <kineticLaw>
          <math xmlns="http://www.w3.org/1998/Math/MathML">
            <apply>
              <times/>
              <ci> cell </ci>
              <apply>
                <ci> Two_sub </ci>
                <ci> V </ci>
                <ci> aconitase </ci>
                <ci> ISC_Grx1r_4Fe_4S </ci>
                <ci> KmA </ci>
                <ci> KmB </ci>
              </apply>
            </apply>
          </math>
                  <listOfParameters>
            <parameter id="V" name="V" value="0.1766"/>
            <parameter id="KmA" name="KmA" value="0.0029"/>
            <parameter id="KmB" name="KmB" value="3.612E-5"/>
          </listOfParameters>
        </kineticLaw>
      </reaction>
      <reaction id="Fum_activation_grx1" metaid="COPASI18"
name="Fum_activation_grx1" reversible="false">
        <annotation>
          <COPASI xmlns="http://www.copasi.org/static/sbml">
            <rdf:RDF xmlns:dcterms="http://purl.org/dc/terms/"
xmlns:rdf="http://www.w3.org/1999/02/22-rdf-syntax-ns#">
              <rdf:Description rdf:about="#COPASI18">
                <dcterms:created>
                  <rdf:Description>
```

```
<dcterms:W3CDTF>2018-07-09T14:19:25Z</dcterms:W3CDTF>
                  </rdf:Description>
                </dcterms:created>
              </rdf:Description>
            </rdf:RDF>
          </COPASI>
        </annotation>
              <listOfReactants>
          <speciesReference species="Fumarase"
stoichiometry="1"/>
          <speciesReference species="ISC_Grx1r_4Fe_4S"
stoichiometry="1"/>
        </listOfReactants>
        <listOfProducts>
          <speciesReference species="fumarase_4Fe_4S"
stoichiometry="1"/>
          <speciesReference species="ISCU" stoichiometry="1"/>
        </listOfProducts>
        <kineticLaw>
          <math xmlns="http://www.w3.org/1998/Math/MathML">
            <apply>
              <times/>
              <ci> cell </ci>
              <apply>
                <ci> Two_sub </ci>
                <ci> V </ci>
                <ci> Fumarase </ci>
                <ci> ISC_Grx1r_4Fe_4S </ci>
                <ci> KmA </ci>
                <ci> KmB </ci>
              </apply>
            </apply>
          </math>
                  <listOfParameters>
            <parameter id="V" name="V" value="0.1766"/>
            <parameter id="KmA" name="KmA" value="0.0025"/>
            <parameter id="KmB" name="KmB" value="3.612E-5"/>
          </listOfParameters>
        </kineticLaw>
      </reaction>
      <reaction id="Grx1_reduction" metaid="COPASI19" name="Grx1
reduction" reversible="false">
        <annotation>
          <COPASI xmlns="http://www.copasi.org/static/sbml">
            <rdf:RDF xmlns:dcterms="http://purl.org/dc/terms/"
xmlns:rdf="http://www.w3.org/1999/02/22-rdf-syntax-ns#">
              <rdf:Description rdf:about="#COPASI19">
                <dcterms:created>
                  <rdf:Description>

<dcterms:W3CDTF>2018-07-09T17:08:39Z</dcterms:W3CDTF>
                  </rdf:Description>
```

```
                </dcterms:created>
              </rdf:Description>
            </rdf:RDF>
          </COPASI>
        </annotation>
              <listOfReactants>
          <speciesReference species="Grx1" stoichiometry="1"/>
          <speciesReference species="T_SH_2" stoichiometry="1"/>
        </listOfReactants>
        <listOfProducts>
          <speciesReference species="Grx1r" stoichiometry="1"/>
          <speciesReference species="TS2" stoichiometry="1"/>
        </listOfProducts>
        <kineticLaw>
          <math xmlns="http://www.w3.org/1998/Math/MathML">
            <apply>
              <times/>
              <ci> cell </ci>
              <ci> k1 </ci>
              <ci> Grx1 </ci>
              <ci> T_SH_2 </ci>
            </apply>
          </math>
                  <listOfParameters>
            <parameter id="k1" name="k1" value="0.0283333"/>
          </listOfParameters>
        </kineticLaw>
      </reaction>
      <reaction id="Grx2_reduction" metaid="COPASI20" name="Grx2
reduction" reversible="false">
        <annotation>
          <COPASI xmlns="http://www.copasi.org/static/sbml">
            <rdf:RDF xmlns:dcterms="http://purl.org/dc/terms/"
xmlns:rdf="http://www.w3.org/1999/02/22-rdf-syntax-ns#">
              <rdf:Description rdf:about="#COPASI20">
                <dcterms:created>
                  <rdf:Description>

<dcterms:W3CDTF>2018-07-09T17:09:14Z</dcterms:W3CDTF>
                  </rdf:Description>
                </dcterms:created>
              </rdf:Description>
            </rdf:RDF>
          </COPASI>
        </annotation>
              <listOfReactants>
          <speciesReference species="Grx2" stoichiometry="1"/>
          <speciesReference species="T_SH_2" stoichiometry="1"/>
        </listOfReactants>
        <listOfProducts>
          <speciesReference species="Grx2r" stoichiometry="1"/>
          <speciesReference species="TS2" stoichiometry="1"/>
```

```
        </listOfProducts>
        <kineticLaw>
          <math xmlns="http://www.w3.org/1998/Math/MathML">
            <apply>
              <times/>
              <ci> cell </ci>
              <ci> k1 </ci>
              <ci> Grx2 </ci>
              <ci> T_SH_2 </ci>
            </apply>
          </math>
                <listOfParameters>
            <parameter id="k1" name="k1" value="0.0183333"/>
          </listOfParameters>
        </kineticLaw>
      </reaction>
      <reaction id="ISCU_grx1_complex" metaid="COPASI21"
name="ISCU-grx1 complex" reversible="false">
        <annotation>
          <COPASI xmlns="http://www.copasi.org/static/sbml">
            <rdf:RDF xmlns:dcterms="http://purl.org/dc/terms/"
xmlns:rdf="http://www.w3.org/1999/02/22-rdf-syntax-ns#">
              <rdf:Description rdf:about="#COPASI21">
                <dcterms:created>
                  <rdf:Description>

<dcterms:W3CDTF>2018-07-11T12:24:27Z</dcterms:W3CDTF>
                  </rdf:Description>
                </dcterms:created>
              </rdf:Description>
            </rdf:RDF>
          </COPASI>
        </annotation>
              <listOfReactants>
          <speciesReference species="ISCU_2Fe_2S"
stoichiometry="1"/>
          <speciesReference species="Grx1r" stoichiometry="1"/>
        </listOfReactants>
        <listOfProducts>
          <speciesReference species="ISC_Grx1r_4Fe_4S"
stoichiometry="1"/>
        </listOfProducts>
        <kineticLaw>
          <math xmlns="http://www.w3.org/1998/Math/MathML">
            <apply>
              <times/>
              <ci> cell </ci>
              <ci> k1 </ci>
              <ci> ISCU_2Fe_2S </ci>
              <ci> Grx1r </ci>
            </apply>
          </math>
```

```
                  <listOfParameters>
            <parameter id="k1" name="k1" value="333.333"/>
          </listOfParameters>
        </kineticLaw>
      </reaction>
      <reaction id="ISCU_grx2_complex" metaid="COPASI22"
name="ISCU-grx2 complex" reversible="false">
        <annotation>
          <COPASI xmlns="http://www.copasi.org/static/sbml">
            <rdf:RDF xmlns:dcterms="http://purl.org/dc/terms/"
xmlns:rdf="http://www.w3.org/1999/02/22-rdf-syntax-ns#">
              <rdf:Description rdf:about="#COPASI22">
                <dcterms:created>
                  <rdf:Description>

<dcterms:W3CDTF>2018-07-11T12:24:58Z</dcterms:W3CDTF>
                  </rdf:Description>
                </dcterms:created>
              </rdf:Description>
            </rdf:RDF>
          </COPASI>
        </annotation>
              <listOfReactants>
          <speciesReference species="ISCU_2Fe_2S"
stoichiometry="1"/>
          <speciesReference species="Grx2r" stoichiometry="1"/>
        </listOfReactants>
        <listOfProducts>
          <speciesReference species="ISC_Grx2r_4Fe_4S"
stoichiometry="1"/>
        </listOfProducts>
        <kineticLaw>
          <math xmlns="http://www.w3.org/1998/Math/MathML">
            <apply>
              <times/>
              <ci> cell </ci>
              <ci> k1 </ci>
              <ci> ISCU_2Fe_2S </ci>
              <ci> Grx2r </ci>
            </apply>
          </math>
                  <listOfParameters>
            <parameter id="k1" name="k1" value="333.33"/>
          </listOfParameters>
        </kineticLaw>
      </reaction>
      <reaction id="Aco_activation_grx2" metaid="COPASI23"
name="Aco_activation_grx2" reversible="false">
        <annotation>
          <COPASI xmlns="http://www.copasi.org/static/sbml">
            <rdf:RDF xmlns:dcterms="http://purl.org/dc/terms/"
xmlns:rdf="http://www.w3.org/1999/02/22-rdf-syntax-ns#">
```

```
              <rdf:Description rdf:about="#COPASI23">
                <dcterms:created>
                  <rdf:Description>

<dcterms:W3CDTF>2018-07-11T12:28:22Z</dcterms:W3CDTF>
                  </rdf:Description>
                </dcterms:created>
              </rdf:Description>
            </rdf:RDF>
          </COPASI>
        </annotation>
              <listOfReactants>
          <speciesReference species="aconitase"
stoichiometry="1"/>
          <speciesReference species="ISC_Grx2r_4Fe_4S"
stoichiometry="1"/>
        </listOfReactants>
        <listOfProducts>
          <speciesReference species="aconitase_4Fe_4S"
stoichiometry="1"/>
          <speciesReference species="ISCU" stoichiometry="1"/>
        </listOfProducts>
        <kineticLaw>
          <math xmlns="http://www.w3.org/1998/Math/MathML">
            <apply>
              <times/>
              <ci> cell </ci>
              <apply>
                <ci> Two_sub </ci>
                <ci> V </ci>
                <ci> aconitase </ci>
                <ci> ISC_Grx2r_4Fe_4S </ci>
                <ci> KmA </ci>
                <ci> KmB </ci>
              </apply>
            </apply>
          </math>
                  <listOfParameters>
            <parameter id="V" name="V" value="0.1766"/>
            <parameter id="KmA" name="KmA" value="0.0029"/>
            <parameter id="KmB" name="KmB" value="3.612E-5"/>
          </listOfParameters>
        </kineticLaw>
      </reaction>
      <reaction id="Fum_activation_grx2" metaid="COPASI24"
name="Fum_activation_grx2" reversible="false">
        <annotation>
          <COPASI xmlns="http://www.copasi.org/static/sbml">
            <rdf:RDF xmlns:dcterms="http://purl.org/dc/terms/"
xmlns:rdf="http://www.w3.org/1999/02/22-rdf-syntax-ns#">
              <rdf:Description rdf:about="#COPASI24">
                <dcterms:created>
```

```
                  <rdf:Description>

<dcterms:W3CDTF>2018-07-11T12:29:02Z</dcterms:W3CDTF>
                  </rdf:Description>
                </dcterms:created>
              </rdf:Description>
            </rdf:RDF>
          </COPASI>
        </annotation>
              <listOfReactants>
          <speciesReference species="Fumarase"
stoichiometry="1"/>
          <speciesReference species="ISC_Grx2r_4Fe_4S"
stoichiometry="1"/>
        </listOfReactants>
        <listOfProducts>
          <speciesReference species="fumarase_4Fe_4S"
stoichiometry="1"/>
          <speciesReference species="ISCU" stoichiometry="1"/>
        </listOfProducts>
        <kineticLaw>
          <math xmlns="http://www.w3.org/1998/Math/MathML">
            <apply>
              <times/>
              <ci> cell </ci>
              <apply>
                <ci> Two_sub </ci>
                <ci> V </ci>
                <ci> Fumarase </ci>
                <ci> ISC_Grx2r_4Fe_4S </ci>
                <ci> KmA </ci>
                <ci> KmB </ci>
              </apply>
            </apply>
          </math>
                  <listOfParameters>
            <parameter id="V" name="V" value="0.1766"/>
            <parameter id="KmA" name="KmA" value="0.0029"/>
            <parameter id="KmB" name="KmB" value="3.612E-5"/>
          </listOfParameters>
        </kineticLaw>
      </reaction>
      <reaction id="Aconitase_O2_" metaid="COPASI25"
name="Aconitase_O2.-" reversible="false">
        <annotation>
          <COPASI xmlns="http://www.copasi.org/static/sbml">
            <rdf:RDF xmlns:dcterms="http://purl.org/dc/terms/"
xmlns:rdf="http://www.w3.org/1999/02/22-rdf-syntax-ns#">
              <rdf:Description rdf:about="#COPASI25">
                <dcterms:created>
                  <rdf:Description>
```

```
<dcterms:W3CDTF>2018-07-11T12:37:44Z</dcterms:W3CDTF>
                  </rdf:Description>
                </dcterms:created>
              </rdf:Description>
            </rdf:RDF>
          </COPASI>
        </annotation>
              <listOfReactants>
          <speciesReference species="O2_" stoichiometry="1"/>
          <speciesReference species="aconitase_4Fe_4S"
stoichiometry="1"/>
        </listOfReactants>
        <listOfProducts>
          <speciesReference species="aconitase_3Fe_4S_"
stoichiometry="1"/>
          <speciesReference species="H2O2" stoichiometry="1"/>
          <speciesReference species="Fe_3" stoichiometry="1"/>
        </listOfProducts>
        <kineticLaw>
          <math xmlns="http://www.w3.org/1998/Math/MathML">
            <apply>
              <times/>
              <ci> cell </ci>
              <ci> k1 </ci>
              <ci> O2_ </ci>
              <ci> aconitase_4Fe_4S </ci>
            </apply>
          </math>
                  <listOfParameters>
            <parameter id="k1" name="k1" value="3.5E-6"/>
          </listOfParameters>
        </kineticLaw>
      </reaction>
      <reaction id="Fumarase_O2_" metaid="COPASI26"
name="Fumarase O2.-" reversible="false">
        <annotation>
          <COPASI xmlns="http://www.copasi.org/static/sbml">
            <rdf:RDF xmlns:dcterms="http://purl.org/dc/terms/"
xmlns:rdf="http://www.w3.org/1999/02/22-rdf-syntax-ns#">
              <rdf:Description rdf:about="#COPASI26">
                <dcterms:created>
                  <rdf:Description>

<dcterms:W3CDTF>2018-07-11T12:45:10Z</dcterms:W3CDTF>
                  </rdf:Description>
                </dcterms:created>
              </rdf:Description>
            </rdf:RDF>
          </COPASI>
        </annotation>
              <listOfReactants>
          <speciesReference species="O2_" stoichiometry="1"/>
```

```
          <speciesReference species="fumarase_4Fe_4S"
stoichiometry="1"/>
        </listOfReactants>
        <listOfProducts>
          <speciesReference species="fumarase_3Fe_4S_"
stoichiometry="1"/>
          <speciesReference species="H2O2" stoichiometry="1"/>
          <speciesReference species="Fe_3" stoichiometry="1"/>
        </listOfProducts>
        <kineticLaw>
          <math xmlns="http://www.w3.org/1998/Math/MathML">
            <apply>
              <times/>
              <ci> cell </ci>
              <ci> k1 </ci>
              <ci> O2_ </ci>
              <ci> fumarase_4Fe_4S </ci>
            </apply>
          </math>
                <listOfParameters>
            <parameter id="k1" name="k1" value="3.5E-6"/>
          </listOfParameters>
        </kineticLaw>
      </reaction>
      <reaction id="Fenton_reaction" metaid="COPASI27"
name="Fenton reaction" reversible="false">
        <annotation>
          <COPASI xmlns="http://www.copasi.org/static/sbml">
            <rdf:RDF xmlns:dcterms="http://purl.org/dc/terms/"
xmlns:rdf="http://www.w3.org/1999/02/22-rdf-syntax-ns#">
              <rdf:Description rdf:about="#COPASI27">
                <dcterms:created>
                  <rdf:Description>

<dcterms:W3CDTF>2018-07-11T12:46:05Z</dcterms:W3CDTF>
                  </rdf:Description>
                </dcterms:created>
              </rdf:Description>
            </rdf:RDF>
          </COPASI>
        </annotation>
              <listOfReactants>
          <speciesReference species="H2O2" stoichiometry="1"/>
          <speciesReference species="Fe_3" stoichiometry="1"/>
        </listOfReactants>
        <listOfProducts>
          <speciesReference species="_OH" stoichiometry="1"/>
          <speciesReference species="OH" stoichiometry="1"/>
          <speciesReference species="Fe_2" stoichiometry="1"/>
        </listOfProducts>
        <kineticLaw>
          <math xmlns="http://www.w3.org/1998/Math/MathML">
```

```
            <apply>
              <times/>
              <ci> cell </ci>
              <ci> k1 </ci>
              <ci> H2O2 </ci>
              <ci> Fe_3 </ci>
            </apply>
          </math>
                <listOfParameters>
            <parameter id="k1" name="k1" value="76"/>
          </listOfParameters>
        </kineticLaw>
      </reaction>
      <reaction id="Fum_activation_Fe_2" metaid="COPASI28"
name="Fum_activation_Fe+2" reversible="false">
        <annotation>
          <COPASI xmlns="http://www.copasi.org/static/sbml">
            <rdf:RDF xmlns:dcterms="http://purl.org/dc/terms/"
xmlns:rdf="http://www.w3.org/1999/02/22-rdf-syntax-ns#">
              <rdf:Description rdf:about="#COPASI28">
                <dcterms:created>
                  <rdf:Description>

<dcterms:W3CDTF>2018-07-11T17:10:23Z</dcterms:W3CDTF>
                  </rdf:Description>
                </dcterms:created>
              </rdf:Description>
            </rdf:RDF>
          </COPASI>
        </annotation>
              <listOfReactants>
          <speciesReference species="fumarase_3Fe_4S_"
stoichiometry="1"/>
          <speciesReference species="Fe_2" stoichiometry="1"/>
        </listOfReactants>
        <listOfProducts>
          <speciesReference species="fumarase_4Fe_4S"
stoichiometry="1"/>
        </listOfProducts>
        <kineticLaw>
          <math xmlns="http://www.w3.org/1998/Math/MathML">
            <apply>
              <times/>
              <ci> cell </ci>
              <ci> k1 </ci>
              <ci> fumarase_3Fe_4S_ </ci>
              <ci> Fe_2 </ci>
            </apply>
          </math>
                <listOfParameters>
            <parameter id="k1" name="k1" value="0.0017"/>
          </listOfParameters>
```

```
        </kineticLaw>
      </reaction>
      <reaction id="Aco_activation_Fe_2" metaid="COPASI29"
name="Aco_activation_Fe+2" reversible="false">
        <annotation>
          <COPASI xmlns="http://www.copasi.org/static/sbml">
            <rdf:RDF xmlns:dcterms="http://purl.org/dc/terms/"
xmlns:rdf="http://www.w3.org/1999/02/22-rdf-syntax-ns#">
              <rdf:Description rdf:about="#COPASI29">
                <dcterms:created>
                  <rdf:Description>

<dcterms:W3CDTF>2018-07-11T17:08:35Z</dcterms:W3CDTF>
                  </rdf:Description>
                </dcterms:created>
              </rdf:Description>
            </rdf:RDF>
          </COPASI>
        </annotation>
              <listOfReactants>
          <speciesReference species="aconitase_3Fe_4S_"
stoichiometry="1"/>
          <speciesReference species="Fe_2" stoichiometry="1"/>
        </listOfReactants>
        <listOfProducts>
          <speciesReference species="aconitase_4Fe_4S"
stoichiometry="1"/>
        </listOfProducts>
        <kineticLaw>
          <math xmlns="http://www.w3.org/1998/Math/MathML">
            <apply>
              <times/>
              <ci> cell </ci>
              <ci> k1 </ci>
              <ci> aconitase_3Fe_4S_ </ci>
              <ci> Fe_2 </ci>
            </apply>
          </math>
                  <listOfParameters>
            <parameter id="k1" name="k1" value="0.0017"/>
          </listOfParameters>
        </kineticLaw>
      </reaction>
      <reaction id="Aco_ONOO" metaid="COPASI30" name="Aco_ONOO-"
reversible="false">
        <annotation>
          <COPASI xmlns="http://www.copasi.org/static/sbml">
            <rdf:RDF xmlns:dcterms="http://purl.org/dc/terms/"
xmlns:rdf="http://www.w3.org/1999/02/22-rdf-syntax-ns#">
              <rdf:Description rdf:about="#COPASI30">
                <dcterms:created>
                  <rdf:Description>
```

```
<dcterms:W3CDTF>2018-07-12T10:35:24Z</dcterms:W3CDTF>
                  </rdf:Description>
                </dcterms:created>
              </rdf:Description>
            </rdf:RDF>
          </COPASI>
        </annotation>
              <listOfReactants>
          <speciesReference species="aconitase_4Fe_4S"
stoichiometry="1"/>
          <speciesReference species="ONOO" stoichiometry="1"/>
        </listOfReactants>
        <listOfProducts>
          <speciesReference species="aconitase_3Fe_4S_"
stoichiometry="1"/>
          <speciesReference species="Fe_3" stoichiometry="1"/>
        </listOfProducts>
        <kineticLaw>
          <math xmlns="http://www.w3.org/1998/Math/MathML">
            <apply>
              <times/>
              <ci> cell </ci>
              <ci> k1 </ci>
              <ci> aconitase_4Fe_4S </ci>
              <ci> ONOO </ci>
            </apply>
          </math>
                  <listOfParameters>
            <parameter id="k1" name="k1" value="140000"/>
          </listOfParameters>
        </kineticLaw>
      </reaction>
      <reaction id="Fum_ONOO" metaid="COPASI31" name="Fum_ONOO-"
reversible="false">
        <annotation>
          <COPASI xmlns="http://www.copasi.org/static/sbml">
            <rdf:RDF xmlns:dcterms="http://purl.org/dc/terms/"
xmlns:rdf="http://www.w3.org/1999/02/22-rdf-syntax-ns#">
              <rdf:Description rdf:about="#COPASI31">
                <dcterms:created>
                  <rdf:Description>

<dcterms:W3CDTF>2018-07-12T10:36:01Z</dcterms:W3CDTF>
                  </rdf:Description>
                </dcterms:created>
              </rdf:Description>
            </rdf:RDF>
          </COPASI>
        </annotation>
              <listOfReactants>
          <speciesReference species="fumarase_4Fe_4S"
```

```
stoichiometry="1"/>
          <speciesReference species="ONOO" stoichiometry="1"/>
        </listOfReactants>
        <listOfProducts>
          <speciesReference species="fumarase_3Fe_4S_"
stoichiometry="1"/>
          <speciesReference species="Fe_3" stoichiometry="1"/>
        </listOfProducts>
        <kineticLaw>
          <math xmlns="http://www.w3.org/1998/Math/MathML">
            <apply>
              <times/>
              <ci> cell </ci>
              <ci> k1 </ci>
              <ci> fumarase_4Fe_4S </ci>
              <ci> ONOO </ci>
            </apply>
          </math>
                  <listOfParameters>
            <parameter id="k1" name="k1" value="140000"/>
          </listOfParameters>
        </kineticLaw>
      </reaction>
    </listOfReactions>
  </model>
</sbml>
```
